# AgBase: a functional genomics resource for agriculture

**DOI:** 10.1186/1471-2164-7-229

**Published:** 2006-09-08

**Authors:** Fiona M McCarthy, Nan Wang, G Bryce Magee, Bindu Nanduri, Mark L Lawrence, Evelyn B Camon, Daniel G Barrell, David P Hill, Mary E Dolan, W Paul Williams, Dawn S Luthe, Susan M Bridges, Shane C Burgess

**Affiliations:** 1 Department of Basic Sciences, College of Veterinary Medicine, Mississippi State University, P.O. Box 1600, Mississippi State, MS 39762, USA; 2 Department of Computer Science and Engineering, Bagley College of Engineering, P.O. Box 9637, Mississippi State University, MS 39762, USA; 3 European Bioinformatics Institute, Wellcome Trust Genome Campus, Hinxton, Cambridge CB10 1SD, UK; 4 Mouse Genome Informatics, The Jackson Laboratory 600 Main Street, Bar Harbor, ME 04609, USA; 5 USDA ARS Corn Host Plant Resistance Research Unit, Box 5367, Mississippi State University, MS 39762, USA; 6 Department of Biochemistry and Molecular Biology, P.O. Box 9650, Mississippi State University, MS 39762, USA; 7 Institute for Digital Biology, Mississippi State University, MS 39762, USA

## Abstract

**Background:**

Many agricultural species and their pathogens have sequenced genomes and more are in progress. Agricultural species provide food, fiber, xenotransplant tissues, biopharmaceuticals and biomedical models. Moreover, many agricultural microorganisms are human zoonoses. However, systems biology from functional genomics data is hindered in agricultural species because agricultural genome sequences have relatively poor structural and functional annotation and agricultural research communities are smaller with limited funding compared to many model organism communities.

**Description:**

To facilitate systems biology in these traditionally agricultural species we have established "AgBase", a curated, web-accessible, public resource  for structural and functional annotation of agricultural genomes. The AgBase database includes a suite of computational tools to use GO annotations. We use standardized nomenclature following the Human Genome Organization Gene Nomenclature guidelines and are currently functionally annotating chicken, cow and sheep gene products using the Gene Ontology (GO). The computational tools we have developed accept and batch process data derived from different public databases (with different accession codes), return all existing GO annotations, provide a list of products without GO annotation, identify potential orthologs, model functional genomics data using GO and assist proteomics analysis of ESTs and EST assemblies. Our journal database helps prevent redundant manual GO curation. We encourage and publicly acknowledge GO annotations from researchers and provide a service for researchers interested in GO and analysis of functional genomics data.

**Conclusion:**

The AgBase database is the first database dedicated to functional genomics and systems biology analysis for agriculturally important species and their pathogens. We use experimental data to improve structural annotation of genomes and to functionally characterize gene products. AgBase is also directly relevant for researchers in fields as diverse as agricultural production, cancer biology, biopharmaceuticals, human health and evolutionary biology. Moreover, the experimental methods and bioinformatics tools we provide are widely applicable to many other species including model organisms.

## Background

The genomes of agriculturally important organisms are sequenced [1-3] or being sequenced [4,5] not only due to their economic importance but also because many are biomedical models [6-10] or zoonotic pathogens and bioterrorism agents [11]. However, after genome sequencing it is critical to identify and demarcate the functional elements in the genome (structural annotation) and to link these genomic elements to biological function (functional annotation). Current genome assemblies have several thousands of gaps, causing bad gene model predictions due to missing exons and splice sites. Statistics for the chicken and cow genomes compared with the human, mouse and rat genomes (Table 1) reveal fundamental problems in genomic structural and functional annotation in livestock genomes. Livestock genomes will always have low build numbers compared with model organisms such as human and mouse and yet they have comparable numbers of genes (UniGene). A relatively large proportion of these genes in these species are electronically predicted. Another problem is that, compared to human and mouse, the chicken and cow have 10-fold fewer ESTs to aid in structural annotation and functional analysis. These statistics, combined with smaller funding bases and resources for manual genome structural annotation, suggest that the human and mouse paradigm for genome structural annotation is unlikely to be successful for agricultural species [12].

**Figure 1:**
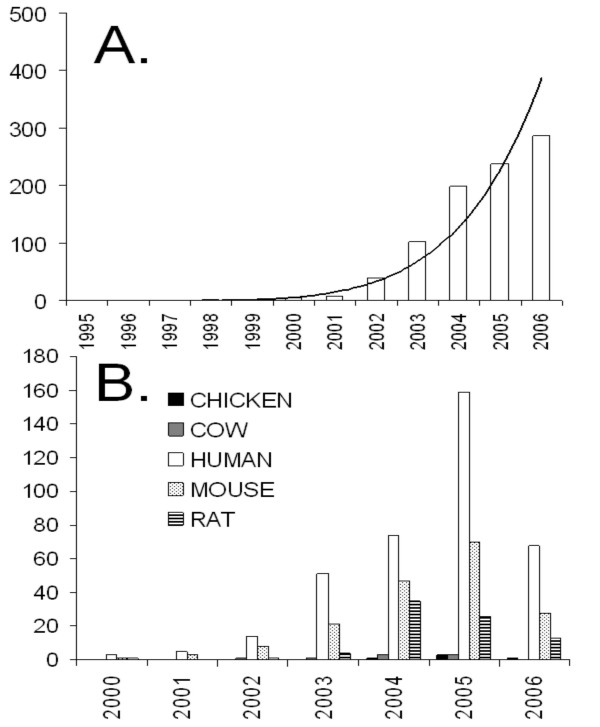
**Papers referencing GO by species**. The number of papers referencing GO, as determined from PubMed (06/09/06). GO annotation has become the accepted standard for functional annotation [13] and its use is growing exponentially (A). Despite this, GO annotation has been minimally used in chicken and cow (B), in part this is because of smaller numbers of livestock researchers, but also using GO annotation in livestock first requires researchers to functionally annotate their own data.

For functional annotation, the GO is the *de facto *standard and its use for modeling microarray and other functional genomics data is growing exponentially [13]. However, this growth in the use of GO is not seen in agricultural species (Figure 1) because of poor GO annotation (Table 1). Gramene [14] and TIGR [15] provide annotations for grasses and microbes, respectively, but most GO annotations for other agriculturally important species are provided by the European Bioinformatics Institute Gene Ontology Annotation project (EBI-GOA) [16]. EBI-GOA annotates proteins in the UniProt Knowledgebase (UniProtKB) only. However, agricultural species have an order of magnitude fewer entries in UniProtKB than human and mouse. Many proteins in agricultural species are still only electronically predicted and reside in the UniProt Archive (UniParc) database, which EBI-GOA does not annotate. Moreover, most GO annotations that do exist for agricultural proteins are "inferred from electronic annotation" (IEA). IEA is usually only applied to broad GO terms and results in very general superficial GO functional information. More detailed functional annotations require expert human curation of experimental evidence, typically from peer-reviewed literature. The rat genome (22) is an interesting example of what can be achieved in GO annotation through a community's concerted effort. The rat genome was published only 8 months prior to the chicken genome. Like the chicken, but unlike human and mouse, rat has relatively few proteins in the UniProtKB and a high proportion of "predicted" proteins in UniParc. However, there are twice as many GO annotations for rat than currently exist for chicken and fewer of these annotations are IEA. Consequently, there is a concomitant growth in rat publications using GO to model microarray and other functional genomics data (Figure 1).

The current state of agricultural genome annotation hinders its utility for systems biology modeling of microarray and other functional genomics datasets. To fully utilize agricultural genome sequence data requires further, computationally accessible, structural and functional annotation. Here we describe "AgBase", a unified resource dedicated to enabling genome-wide structural and functional annotation and modeling of microarray and other functional genomics data in agricultural species. AgBase integrates structural and functional annotations and provides tools in an easy-to-use pipeline, allowing agricultural and biomedical researchers to rapidly and effectively model and derive biological significance from microarray and other functional genomics datasets.

## Construction and content

The AgBase server is a dual Xeon 3.0 processor with a 800 Mhz FSB, 4 GB of Ram and five 146 GB hard drives in a RAID-5 configuration. The operating system is Windows 2000 Server. AgBase has a dedicated tape backup system with a total storage capacity of 3.2 TB native and 6.4 TB compressed. The backup software is Veritas Netback. A full backup is done each weekend, an archive backup once a month, and incremental backups nightly.

AgBase is implemented using the mySQL 4.1 database management system, NCBI Blast, and scripts written in Perl CGI. The schema is a protein centric design that is an adaptation of the Chado schema with extensions to accommodate storage of expressed peptide sequence tags (ePSTs). The entity relationship (ER) model for primary objects in the database for each protein is given as supplementary data [see Additional file 1]. A separate schema is implemented for ePST data. Data that is generated in-house includes AgBase GO annotations, the AgBase gene association files and ePSTs. External data that is integrated into the database includes the Gene Ontology, the UniProt database, EBI-GOA and the NCBI Entrez Taxonomy.

The GO annotations are generated by manual curation of the literature and by sequence similarity (GO evidence code ISS) using the GOanna tool followed by manual inspection of the alignments that are produced. AgBase biocurators are trained in a GO curation course that is held periodically. All literature-based AgBase GO annotations are quality checked to GO Consortium standards. The ePSTs are generated using a proteogenomic mapping pipeline implemented in Perl. The pipeline integrates information from experimental proteomics experiments and annotated genomes. Results are visualized using the Apollo genome browser to allow curation by scientists. Each ePST is quality checked by AgBase Biocurators. The generation of ePSTs is discussed in the experimental structural annotation section below.

Users can access protein information by protein name, gene name, GO term, taxon, a variety of accession numbers, or via BLAST searches. The AgBase tools also access the AgBase database. AgBase is updated from external sources every three months and locally generated data is loaded as it is generated. Gene association files of gene products annotated by AgBase are accessible in a tab-delimited format to facilitate data exchange.

We have purposely followed the paradigm of multi-species databases suggested by Stein [17] and the Reactome database [18] and are currently focused on plants and animals whose genomes are, or will be, sequenced and microbial pathogens and parasites that have significant economic impact on agricultural production and zoonotic disease. AgBase has four main aims (discussed in detail below): (1) to provide experimentally derived structural annotations of agricultural genomes; (2) to provide highly curated, GO functional annotations; (3) to promote the use of standardized nomenclature in agricultural species; (4) to develop computational pipelines for processing and using structural and functional annotations.

## Utility

The AgBase database is intended as a resource to assist functional genomics in agricultural species and the tools provided support analysis of large scale datasets. To this end, we provide both experimentally derived structural annotation and functional data in a unified resource. While agriculturally important organisms may have other resources that provide structural annotation or GO annotations, AgBase is unique because (1) the structural data provided is experimentally derived; (2) the structural and functional data is provided from a unified resource; and (3) tools for analysis of this data are freely available via AgBase. The AgBase interface allows users to search for information in several ways. The Text Search performs an exact substring search on the selected database. To facilitate data sharing, searching based on commonly used accession numbers and identifiers is supported in addition to BLAST searches. Multiple query searches are also available.

## Discussion

### Experimental structural annotation

The use of experimental data for genome annotation is critical for conclusive identification of the functional sequences within genomes, accurate description of intron/exon structures and determination of the potential products from each gene in different tissues and cellular states [19]. Through AgBase we make available improved structural annotation of agriculturally important genomes from experimental confirmation of electronically predicted proteins/open reading frames, especially via proteogenomic mapping [19-23].

Proteogenomic mapping generates expressed peptide sequence tags (ePSTs) [23]. These ePSTs are derived by identifying novel protein fragments through proteomics, aligning these to the genome sequence and extending to the nearest 3' stop codon. We have used the proteogenomic mapping pipeline to generate ePSTs for a prokaryote (*Pasteurella multocida*) and a eukaryote (chicken). *P. multocida*, or chicken "fowl cholera", is a bovine respiratory disease pathogen and human zoonosis. Although the *P. multocida *genome was sequenced in 2001 [24] and is considered well annotated, our proteogenomic pipeline identified 202 ePSTs that had identifiable methionine start codons [see Additional file 2]. One of these is a 130 amino acid ePST that was identified by six different peptides and is located in a 704 bp intergenic region between accA and guaA in the Pm70 genome [see Additional file 3]. The ePST has 60% identity and 74% similarity at the protein level with the 114 amino acid hypothetical protein HD_1218 (Genbank accession AAP96060) from *Haemophilus ducreyi *(a major cause of human genital ulcer disease [chancroid] in humans). A database of ePSTs identified from chicken and *P. multocida *is publicly accessible via the proteogenomics link on the AgBase homepage. The ePST database is fully searchable either by text or Blast searching. Text-searchable fields include taxonID, genome build, chromosome or chromosomal location. Public submissions to the ePST database are cited by submitter name.

Generating ePSTs is time consuming and labor intensive. To facilitate structural annotation we have developed a proteogenomic mapping pipeline for generation of ePSTs (available from AgBase by request). The pipeline for prokaryotes currently includes a visualization component (we currently use Apollo [25]) that allows the researcher to view the ePSTs in context in the genome. In eukaryote genomes it is possible that the extension is carried beyond a splice signal producing an ePST that includes intronic DNA. We are currently in the process of extending the pipeline to detect splice signals and to show alignments with ESTs in the visualizations to address this shortcoming.

To ensure that structural data is based on high quality proteomics identifications, we have developed a method for assigning probabilities to mass spectral identifications during proteogenomic mapping [26]. Assigning probabilities to mass spectral identifications is important because one issue associated with tandem mass spectral searching against databases is false positive and false negative peptide identifications. Moreover, all of our proteomics data is submitted to the PRIDE database [27]. Mass spectrometry data submitted to PRIDE is further curated for inclusion in UniProtKB, where it is available for uploading into genome browsers, for example Ensembl [28]. To add value to the structural annotations provided by AgBase and enhance biological modeling, we have also developed methods and tools for assigning GO annotations (see below).

### Functional annotation

Many gene products from agriculturally important organisms have no GO annotation. Practically, this means that experimentalists working with these species must provide their own GO annotations if they wish to use GO to model their microarray and other functional genomics data. While those best qualified to functionally annotate a gene product may be those who work directly with it [29], few experimentalists can devote the time and resources needed to learn the intricacies of GO biocuration. To facilitate functional modeling in agricultural organisms, we are actively GO annotating chicken, cow, sheep and catfish gene products.

While EBI-GOA uses an electronic mapping strategy to rapidly provide GO annotations for a large number of gene products, these are IEA mappings that rely on curated information from SwissProt, InterPro and the Enzyme Commission (EC) databases [16]. Many agricultural gene products are 'predicted' products based on gene prediction algorithms (Table 1) and do not exist in these curated databases. However, GO annotation can be assigned based on human interpretation of sequence and/or structural similarities (ISS) with well-studied and already GO-annotated gene products. By definition, such gene products can only be annotated to ISS or IEA since they have no experimental functional data as yet.

Our GO annotation strategy first provides *breadth *by focusing on the large proportion of gene products that currently exist in the UniParc database and have no GO annotation. Since predicted proteins represent approximately half of the gene products from newly sequenced genomes (Table 1.), being able to provide GO annotations for these gene products complements the GO annotations provided by EBI-GOA and dramatically improves our ability to model functional genomics data. We are doing a "first-pass" ISS annotation of chicken, cow and sheep gene products that currently have no GO annotation (using manual inspection of BLAST alignments and, where possible, established orthology). In addition, we have developed DDF-MudPIT [30], a high-throughput, proteomics-based method that simultaneously confirms expression and experimentally determines the cellular component of gene products [30]. We next provide finer and more precise functional annotations (i.e. improved GO *depth*) by curating literature. All of our GO annotations are prioritized based on our experimental needs. One example is our recent proteomics model of B-cell development in the chicken bursa of Fabricius [23]. Initially we were hampered because few chicken proteins had any GO functional annotation. We annotated 142 chicken proteins, including curation of 24 PubMed articles. These GO annotations were used to refine cell differentiation, proliferation and cell death modeling in the developing bursa.

**Figure 2:**
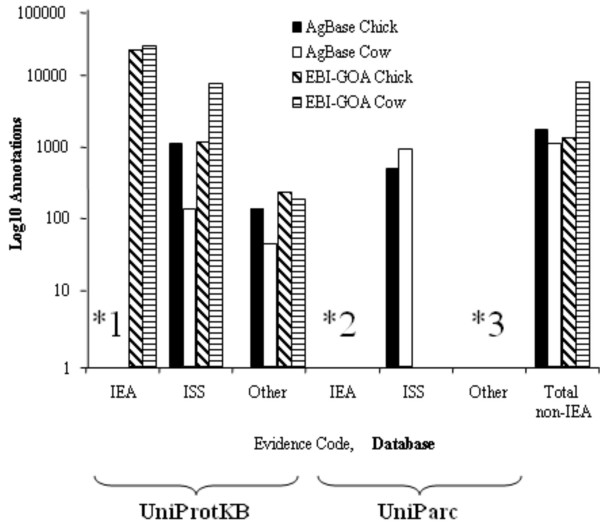
**A comparison of chicken and cow GO annotations from AgBase and EBI-GOA**. We are currently focused on providing GO annotations for chicken and cow gene products and we collaborate with EBI-GOA to provide a combined GO gene association file for each of these species. The number of GO annotations for chicken and cow is represented here based on GO evidence code; details about the GO evidence codes can also be found on the GO Consortium homepage [37]. (1) Unlike EBI-GOA, AgBase does not currently annotate to IEA. (2) In newly sequenced genomes, such as cow and chicken, a large proportion of gene products are not represented in the UniProtKB database (Table 1) and are not annotated by EBI-GOA. To complement the EBI-GOA annotation effort and provide breadth of coverage, we identify the expression of these 'predicted' gene products *in vivo *and, where possible, provide GO annotations. (3) By definition, there is no published literature for these 'predicted' proteins and they can only be GO annotated using either IEA or ISS.

**Figure 3:**
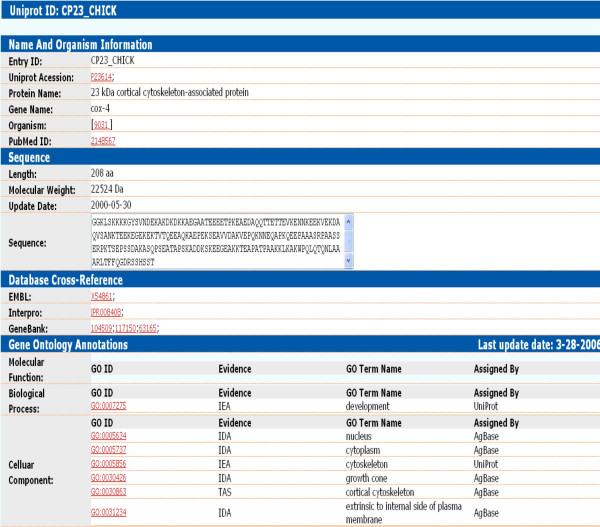
**The AgBase protein detail page**. The AgBase protein detail page shows proteins and their GO annotation. The GO annotation terms are interactive links and the source of the GO annotation is acknowledged. Protein sequence is displayed in a text accessible window and where possible, links to other databases are cross-referenced.

To date (02/15/2006) we have provided GO annotations for chicken, cow, sheep and channel catfish (Table 2). For evidence codes other than IEA (which we do not do), in our first nine months MSU-AgBase made a comparable number of GO associations to chicken as EBI-GOA (Figure 2). Our biocurators collaborate with those at EBI-GOA to provide a single, publicly accessible GO annotation file for both chicken and cow. AgBase-derived GO annotations to gene products that exist in UniProtKB are incorporated with EBI-GOA annotations and added to UniProtKB. For completeness, in addition to our own GO annotations, we include EBI-GOA derived GO annotations (including IEA) in AgBase. The source of these annotations is shown in the protein detail page (Figure 3). Currently AgBase is the only source of GO annotations for UniParc agricultural proteins.

We actively educate, encourage and seek out researchers in the scientific communities to contribute their own GO annotations. We help these researchers properly format their annotations and they are acknowledged for their annotations on the protein detail page for the gene product in AgBase. As public annotations are submitted to AgBase, research-directed GO annotations from the research community will be acknowledged on the protein detail page. We will also supply maize gene product annotations to Gramene [31] and MaizeGDB [32]. To avoid duplication of effort in literature curation, we developed a journal database (JDB) based on PubMed identity number (PMID). JDB tracks all PubMed articles used as a source for manual GO annotations. The JDB will aid collaborative GO annotation as it can be used for quality control for GO annotations among interested groups. Non-biocurators may access the JDB as a guest user.

### Nomenclature

While the GO does not specifically deal with gene nomenclature, unified and unique nomenclature for orthologous genes is essential. Where possible, chicken genes will be assigned nomenclature based on orthologous human nomenclature [33]. We use human orthologs to provide standardized gene symbols to chicken and cow gene products during the process of making GO associations. A chicken gene nomenclature committee is at a formative stage; as yet, no corresponding committee exists for cow.

### Tool development

**Figure 4:**
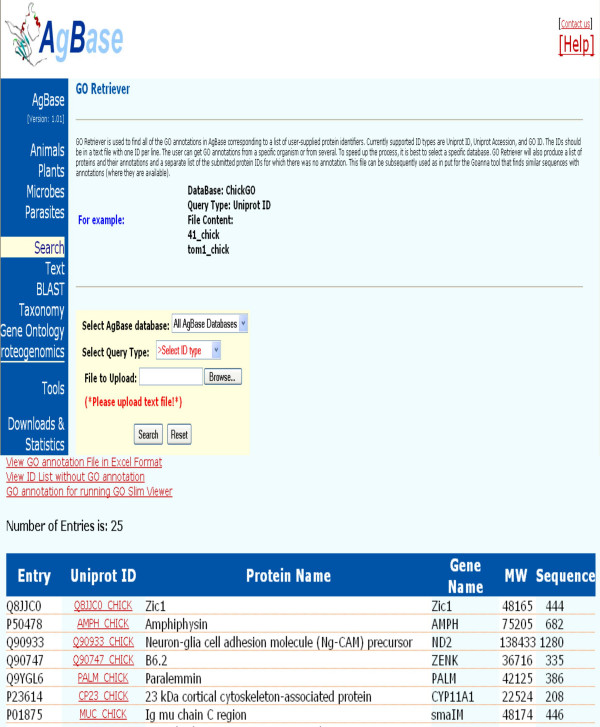
***GORetriever***. *GORetriever *takes a list of accession numbers or IDs and fetches the existing GO annotation for these products. A list of IDs for which there is currently no GO annotation is also returned and may be used as input for *GOanna *(Figure 5). An example of a chicken protein and its corresponding matches is shown.

**Figure 5:**
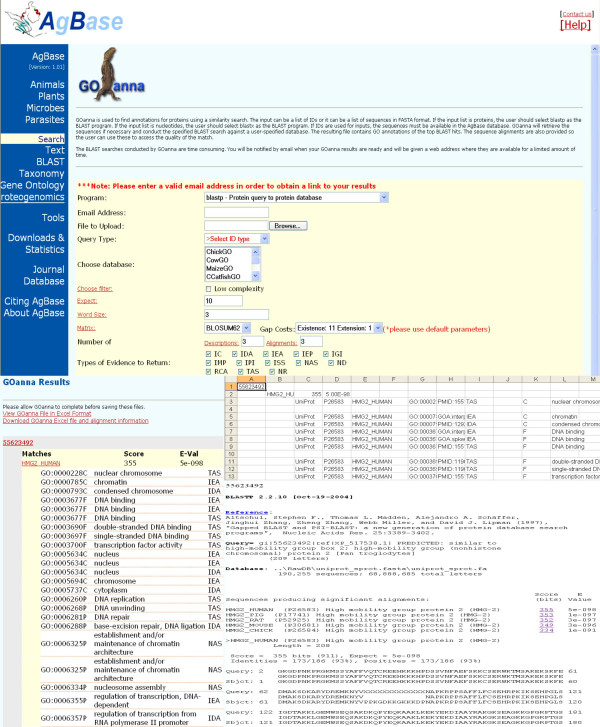
***GOanna***. *GOanna *allows a user to make GO annotations based on sequence similarity. The user inputs a file of IDs or sequences and the tool does a Blast search against a user-specified database of GO annotated gene products using user-defined parameters. The output is shown both at the web interface and as a downloadable file that contains hyperlinks to the BlastP alignments.

**Figure 6:**
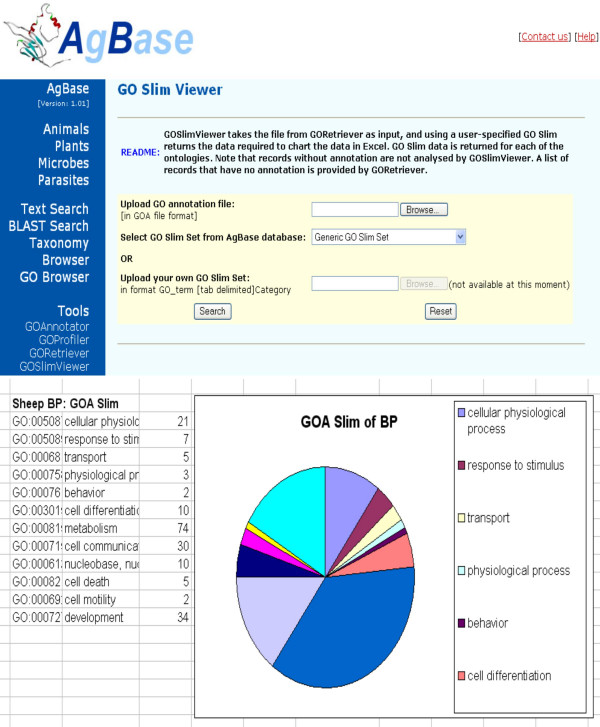
***GOSlimViewer***. *GOSlimViewer *takes a list of list of GO numbers generated from the GO Retriever program (A) and using a user-defined slim, creates an Excel compatible file that can be used for visualization of the results (B).

We have developed freely available computational tools to help researchers use the GO to derive biological significance from their microarray and other functional genomics data. These tools are designed as part of an integrated pipeline to batch process input. In order to improve interoperability with different types of data, the tools accept several input formats. Our GO annotation suite of tools is available online via the Tools link at the AgBase homepage. These tools can also be used for non-agricultural organisms, including newly sequenced species and those without complete genome sequence available. The steps to analyze a microarray or other functional genomics dataset are:

1. Enter the list of accession numbers into *GORetriever *to return all existing GO annotations available for that dataset (Figure 4). *GORetriever *also provides a list of proteins without GO annotation. The researcher then enters this second list into the *GOanna *tool.

2. *GOanna *accepts either a list of IDs or a user defined file of sequences in FASTA format and does a Blast search against databases containing only annotated proteins. The user can choose the number of Blast hits to retrieve per query and set the "Evalue" threshold (NCBI thresholds are the default). The *GOanna *output file contains hyperlinks that direct the user to the original Blast alignment (Figure 5) so that the user can make their own value judgments for their ISS GO annotations.

3. After ISS annotation, the user may choose to annotate the data further by curating published literature. We provide advice on GO annotation and are developing a mechanism for researchers to be publicly acknowledged for GO annotations they submit to AgBase.

4. After annotation, the researcher can then use *GOSlimViewer *to summarize GO data for each of the three ontologies in chart form (Figure 6). *GOSlimViewer *accepts a text-based file created from the above pipeline as input and, using a user-specified GO Slim, returns a text simple text file. This file can be opened and charted in Excel to obtain publication quality figures.

We are committed to developing tools and pipelines to maximize the payoff gained from expensive high-throughput microarray and other functional genomics experiments. We have designed tools that may be applied across a diverse range of species, including microbes, parasites, viruses, plants and animals. For example, the same tools used to model B-cell development in the chicken allowed us to formulate experimental models for disease resistance in maize. We identified 1,522 unique proteins from the developing maize rachis (cob) using a combination of MudPIT and 2-D electrophoresis. In addition, rachis proteins from *Aspergillus flavus *resistant and susceptible lines were compared by differential gel electrophoresis: seventy-three proteins that were more abundant in resistant lines (1.5-fold or greater). Using the tools described above we divided these over-expressed proteins into four categories: abiotic stress proteins; antioxidant enzymes; enzymes in the phenylpropanoid pathway leading to flavonoid and lignin biosynthesis; and proteins with various other metabolic functions. Analyses of these data will help us formulate testable hypotheses regarding the role of the maize rachis in resistance to *A. flavus *infection and aflatoxin accumulation.

Finally, we also develop tools for agriculturally important species that do not yet (or may never have) their genomes sequenced. Researchers working with such species often rely on ESTs and EST assemblies for functional analysis. However, most ESTs (and microarrays derived from these) are not associated with GO annotation. *GOanna *accepts FASTA files and can be used associate GO function with ESTs. Another tool to enable EST modeling is the *ProtIDer *tool (freely available by request to AgBase). *ProtIDer *is a homology-based search program that provides an automated pipeline for the proteomic analysis of ESTs and EST assemblies from TIGR [34-36]. The *ProtIDer *tool compares EST assemblies or singleton ESTs to the UniProtKB and uses high-matching proteins to correct sequencing errors and to annotate the sequence. We have tested *ProtIDer *using data obtained from channel catfish, an organism which currently has only 1,108 protein records in the NRPD, but has 45,622 ESTs available from the dbEST (03/20/06). Tandem mass spectra obtained from channel catfish ovary cells was used to search against three databases: all catfish entries in the NRPD (cfNRPD); a database of highly homologous proteins (hpDB) that come from NRPD as a result of TBLASTN-searching the NRPD with all catfish ESTs generated using *ProtIDer*; and the ESTs themselves translated in all 6 frames (cfESTDB). We identified 1001 proteins and ESTs [see Additional file 4]: 10 from cfNRPD (4 were ribosomal proteins); 48 from the hpDB (only 5 of which were ribosomal) and 962 from cfESTDB. These approaches provide complementary annotation information. Not all of the cfNRPD entries are yet represented in the EST databases; the hpDB allowed us to identify highly conserved proteins and searching the cfESTDB directly indicates ESTs that may be translated. When we used ISS to the hpDB to make GO associations to catfish ESTs we found that the GO terms were distributed over the cellular component, although the biological process had a larger proportion of gene products annotated to response to stimulus and cell communication. From this initial data we will focus on modeling cell communication pathways in developing channel catfish ovary cells.

### Future developments

We are building upon the tools and resources already available at AgBase. The proteogenomics pipeline is being extended to allow more informative visualization of ePSTs in context within the genome and alignment with ESTs and orthologous sequences from other organisms. We will continue to generate ePSTs for newly sequenced agricultural genomes and will also continue to add GO annotations for agriculturally important organisms. We are working to improve the representation of agricultural gene products in the UniProtKB and a tangible example of this is the recent addition of experimentally confirmed chicken 'predicted' gene proteins [23] added to the UniProtKB database.

## Conclusion

We have improved the structural annotation of agriculturally important genomes by experimentally confirming 8,704 predicted proteins in chicken and cow (PRIDE submissions numbers pending) and 723 ePSTs from chicken and *P. multocida*. In our first nine months (04/22/05–03/20/06) we have provided 5,762 new GO annotations to 759 proteins from five different species. While most of our GO annotations are ISS (97%), we have also manually curated 42 PubMed references. We have developed a suite of tools to associate GO annotation with experimental data and to provide higher-order summaries of the data, and a tool to aid EST analysis. Users external to MSU account for more than one third of the hits recorded at the AgBase website.

## Availability and requirements

Access to the AgBase databases is via  and access to data is unrestricted. The tools we have developed are either freely available online at AgBase or by contacting us via the link provided at the AgBase website. The help pages provide information about how to use these tools or technical support can be obtained directly by contacting us. AgBase is an on-going project and interaction with the user community is vital for its success. We encourage the submission of data, correction of errors, and suggestions for making AgBase of greater use, including ideas for new computational tools. Our biocurators make every effort to maintain data integrity by linking data with researchers, references and methods.

## Authors' contributions

FMM developed the GO associations, assisted with the conception and design of the AgBase databases and website, analyzed and interpreted channel catfish data and drafted the manuscript. NW developed the GO and ePSTs databases, developed and implemented the AgBase website, assisted with the conception of the AgBase databases and contributed to tool development. GBM developed the JDB and contributed to tool development. BN designed, analyzed and interpreted the *P. multocida *data, assisted with concepts in tool development and critically reviewed the manuscript. MLL designed, analyzed and interpreted the *P. multocida *data and critically reviewed the manuscript. EBC provided assistance and quality assessment of gene associations and assisted with making AgBase data publicly available. DGB provided tools and strategies for making the initial gene associations and provided quality assurance. DPH provided GO training, mentoring and quality assurance of AgBase GO annotations. MED developed computer strategies for mapping GO terms to chicken gene products and developed the initial ChickGO database. WPW and DSL designed the maize experiments, and analyzed and interpreted the maize data. DSL also contributed to development of the manuscript. SMB developed the conception and design of the AgBase databases and website, assisted with concepts in tool development, analyzed and interpreted channel catfish and maize data and critically reviewed the manuscript. SCB developed the conception and design of the AgBase databases and website, developed concepts for the bioinformatics tools, analyzed and interpreted channel catfish and *P. heamolytica *data and critically reviewed the manuscript.

## Supplementary Material

Additional File 1 **Entity relationship (ER) model of the AgBase database**. The AgBase schema design is protein centric. In addition to the taxonomy identifier, sequence, and GO annotations, mappings to number of identifiers are maintained for each protein. Click here for file

Additional File 2 **ePSTs identified from *P. multocida *using the proteogenomic pipeline developed at AgBase**. *P. multocida *Pm70 ePSTs identified by proteogenomic mapping. For all 202 ePSTS the original MS peptide and its length, the extended ePST and its length as well as the genome start and end positions of the ePST are shown. Click here for file

Additional File 3 **Location of ePST47 in *P. multocida *Pm70 genome**. Region of PM70 genome coding for accA and guaA genes and the 5' end of PMO291 (A). The position in genome is on the left hand side; DNA sequence is shown above; amino acid sequence below; direction of reading frames shown by arrows. The ePST47 is located in the intergenic region between accA and guaA. The figure is taken directly from NCBI nucleotide database graph view with the accession number NC_002663. An expanded view of intergenic region between accA and guaA including ePST47, which runs in the opposite direction to accA, guaA and PMO291 (arrowed) is also shown (B). The overlapping peptides used to identify ePST47 are indicated using bold type (PRIDE accession number pending). This region of the PM70 genome also has 60% identity (* indicates identical nucleotides) and 74% similarity at the protein level with the *Haemophilus ducreyi *hypothetical protein HD_1218 (Genbank accession AAP96060). Click here for file

Additional File 4 **Channel catfish proteins and ESTs identified using the *ProtIDer *tool**. The *ProtIDer *tool is designed for use with species that have large numbers of ESTs but few proteins in the NRPD. *ProtIDer *matches ESTs and EST assemblies to highly homologous proteins from other species by TBLASTN-searching the NRPD with all catfish ESTs. For example, the channel catfish genome sequence is not available and there are only 1,108 NRPD entries for this species. When we analyzed channel catfish ovary tissue we were only able to identify 10 proteins from NRPD. Using *ProtIDer *to create a database of highly homologous proteins resulted in a five-fold increase in the number of proteins identified in this experiment. The proteins identified from the channel catfish NRPD database (cfNRPD), highly homologous database (hpDB) and EST databases are shown here. The *ProtIDer *tool is available from AgBase upon request. Click here for file

## Figures and Tables

**Table 1:** Comparison of human, mouse, rat, chicken and bovine genome statistics.

**Species**	**Genome Build**	**Genes (UniGene)**	**ESTs**
H. sapiens	36.1	86 803	7 741 746
M. musculus	36.1	67 096	4 719 380
R. norvegicus	3.4	51 564	871 147
G. gallus	1.1	30 470	588 288
B. taurus	2.1	41 986	1 039 059

**Species**	**No. Proteins (NRPD)**	**No. Proteins (UniProtKB)**	**% 'Predicted' Proteins**

H. sapiens	299 863	73 932	5.9
M. musculus	184 110	61 537	15.5
R. norvegicus	52 857	14 885	29.9
G. gallus	29 763	7 291	47.9
B. taurus	52 425	10 059	57.1

**Species**	**All GO Associations**	**Non-IEA Associations**	**% IEA Associations**

H. sapiens	266 785	50 612	81
M. musculus	327 082	517 032	60.2
R. norvegicus	73 783	10 285	86.1
G. gallus	29 963	3 058	89.8
B. taurus	39 832	9 484	76.2

Current annotation statistics for selected genomes (15/03/06) are shown. The build number was obtained from NCBI, the estimated number of gene products is based on UniGene numbers [38] and EST numbers are obtained from ESTdb. The number of proteins in the UniProtKB database is under represented for agricultural species. To estimate the proportion of predicted genes in the genome, the number of gene predictions is expressed as a percentage of the total of number of genes both predicted and from UniGene. GO statistics are obtained from GO association files using *GOProfiler *(available from AgBase).

**Table 2:** AgBase GO annotations by species and evidence code.

**Species**	**GO Associations**	**No. Proteins Annotated**	**UniParc Proteins Annotated (ISS)**	**No. Papers Curated**
Chicken	1 007	142	80	24
Cow	4 411	382	4	13
Sheep	316	61	0	0
Channel Catfish	19	3	0	2

We aim to increase the coverage of GO annotations in agriculturally important species and we are currently GO annotating chicken, cow, sheep and channel catfish. To improve GO coverage, we determine which proteins currently have no GO annotations and use *GOanna *to do a 'first-pass' annotation based on sequence homology (ISS). We have also provided GO annotations via literature curation for chicken, cow and channel catfish. We do not currently provide IEA annotations.
