# Supplementary material for: AgBase: a functional genomics resource for agriculture
Source: BMC Genomics. 2006 Sep 8;7:229. doi: 10.1186/1471-2164-7-229 (PMC1618847; doi:10.1186/1471-2164-7-229)
Supplement: Additional File 1 — Entity relationship (ER) model of the AgBase database. The AgBase schema design is protein centric. In addition to the taxonomy identifier, sequence, and GO annotations, mappings to number of identifiers are maintained for each protein. [file 1471-2164-7-229-S1.pdf]

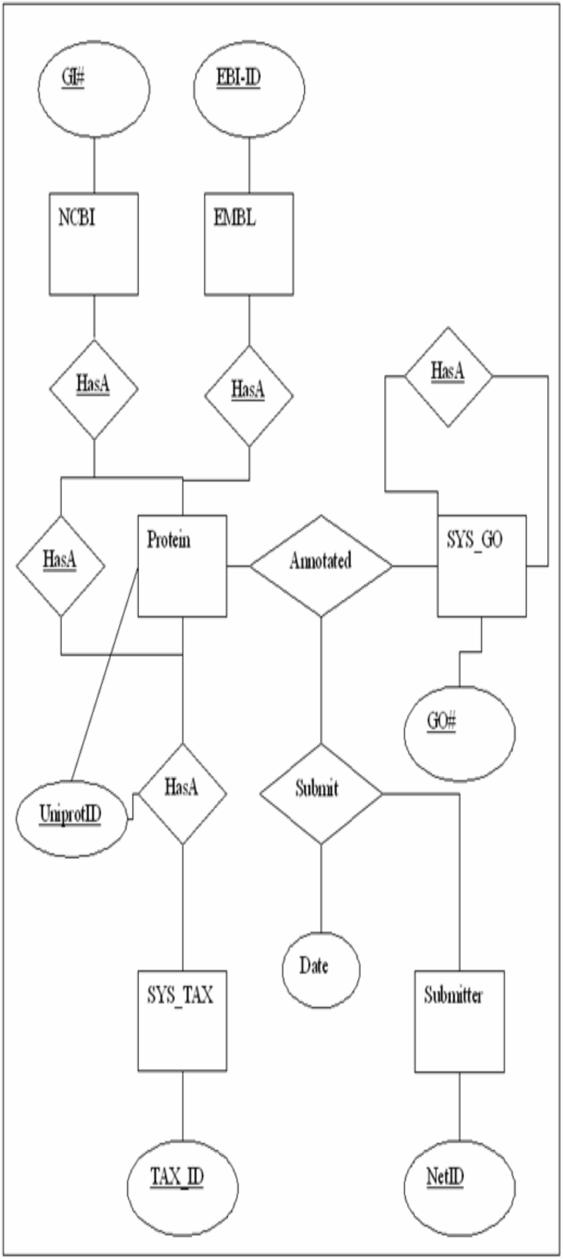

Entity Name	Attributes
Protein	(UniProt_ID, Protein_Name, Specie, SEQ, ...)
GO	(GO_NUM,Description, Type, ...)
Taxonomy	(TAX_ID, Science_Name, Common_Name, ...)
Submitter	(Net_ID, Name, Password, Dep, Email, ...)

Relationship Name	Attributes	Entities
Associated UniProtID	(UniProt_ID, AssociateU_ID)	UniprotID←→UniProtID
Associated EMBLID	(UniProt_ID, AssociateEMBL_ID)	UniProtID←→EMBIID
Associated GI	(UniProt_ID, AssociateGI_NUM)	UniProtID←→GI
Annotated	(UniProt_ID, GO_NUM, Evidence_Code From, Evalue)	Protein ←→GO
Submit	(Submitter, Submit_date, Uniprot_ID, GO_NUM, Evidence_Code)	Protein ←→GO←→Author
HasTAX	(UniProt_ID, TAX_ID)	Protein←→Taxonomy
HasParents	(GO_NUM, **Parent_ID**)	GO←→GO
