# Supplementary material for: AgBase: a functional genomics resource for agriculture
Source: BMC Genomics. 2006 Sep 8;7:229. doi: 10.1186/1471-2164-7-229 (PMC1618847; doi:10.1186/1471-2164-7-229)
Supplement: Additional File 2 — ePSTs identified from P. multocida using the proteogenomic pipeline developed at AgBase. P. multocida Pm70 ePSTs identified by proteogenomic mapping. For all 202 ePSTS the original MS peptide and its length, the extended ePST and its length as well as the genome start and end positions of the ePST are shown. [file 1471-2164-7-229-S2.pdf]

ePST	MS-peptide	Length	ORF with Met start	ORF length	Start	End
1	FSFFTPHFFNTSPSVLSSNAASK	23	MARRISSSRPITGSSLPLSARAVKSIVYFSNAWRLSSAFGSFTFCPP	150	1911359	1910909
2	GFQCPHRHTIVVSDDHFDLICFRAR	25	MCFRGFQCPHRHTIVVSDDHFDLICFRAR	30	1222058	1221968
3	QMLAGSRDILMSAMVRSPMAQHR	23	MCLIISQMIFSGVRQMLAGSRDILMSAMVRSPMAQHR	38	803075	803188
4	RNTVGMIRSGYYLITMSFGK	20	MDFVFLLNNSIVNYWGFRIVSKRNTVGMIRSGYYLITMSFGKKMMR	47	957617	957476
5	LKVVIVPVGNILYLKTAFDAK	21	MDMKLIISDKIVDKLKKKHSVSINEIYECISNIDGKLIEDTAEEHKTIPP	97	1321688	1321397
6	KTMHQRVSFAR	11	MISVAVLISERKTMHQRVSFARIRWKNTAHFILFFSISFSSSLRNHNG	58	588108	588281
7	IEPCVITTLSVKVTTLLSMSK	21	MITLLALMRIEPCVITTLSVKVTTLLSMSKDWLSETTLIGCFVSLFKAK	77	197682	197912
8	LMISAAVPCIGALMATRSAALR	22	MIVRKCSILSVRTSKTNSSCTCMIILMSGCPAISRSKVIIARLMISAAV	86	1256847	1256589
9	KPLVQK	6	MKGKSLSTKVTSLTMRNGRRKPLVQKPPSHTA	33	498064	497965
10	HIYCLMVPPPCWWIKWNK	18	MKHIYCLMVPPPCWWIKWNKWCV	24	1621633	1621561
11	RRMCHSCSIIK	11	MKKQCSYSHRQKKKKRRMCHSCSIIKIMMFR	32	592816	592911
12	TIYFIMCKKMEK	12	MKKTIYFIMCKKMEKEPLMLSLLKKSWQN	30	262537	262447
13	MKQDALCLKR	10	MKQDALCLKRSMIYSI	17	736606	736656
14	RCFMICVK	8	MKRALRRCFMICVKHEGTNKRYRR	25	500324	500398
15	YVKLICELTK	10	MKRYVKLICELTKNLSFCIFVKHGIG	27	1090953	1090872
16	QDQACITLKSIQVVSMMSIYTK	22	MLANFKSCRQDQACITLKSIQVVSMMSIYTKSGSYRSQKALHRVMN	58	1894607	1894780
17	PSLLHILLTSHFVRQCAK	18	MLEKKARPSLLHILLTSHFVRQCAKLIKRTPNQAINQQVHPSYCVRQ	53	1725914	1725755
18	NMMSKPLLSVIAR	13	MLFVKKQYKVLQKNMMSKPLLSVIARAVP	30	1125410	1125499
19	MLITLVKLSMK	11	MLITLVKLSMK	12	308038	308073
20	YMWQPFMLCAQMVWPFLPMNR	21	MLKGKKPPVKSSVVMFIAMTQPQQRYMWQPFMLCAQMVWPFLPM	48	1441752	1441608
21	KTSRVVISSQLMR	13	MLNKNGCTDKNRIPQTARKTSRVVISSQLMRKRFQINQ	39	384052	384168
22	HRICIFCGIAIIHTIDFRR	19	MLNNISIIQLMIKHRICIFCGIAIIHTIDFRRF	34	2227320	2227218
23	LKQTLQAQLAK	11	MLNPKKIEQVIQQIQDSLPQGIKDLGQDAEAKLKQTLQAQLAKLDLV	84	1582305	1582556
24	KIEQVIQQIQDSLPQGIK	18	MLNPKKIEQVIQQIQDSLPQGIKDLGQDAEAKLKQTLQAQLAKLDLV	84	1582305	1582556
25	KIEQVIQQIQDSLPQGIKDLGQDAEAK	27	MLNPKKIEQVIQQIQDSLPQGIKDLGQDAEAKLKQTLQAQLAKLDLV	84	1582305	1582556
26	MHALSALMK	9	MLSLSKMHALSALMKIPMQN	21	1508804	1508741
27	ARLRMSSMPK	10	MLSSLLINCCVKARLRMSSMPKC	24	1531788	1531859
28	LRLVSNMPRIMLLR	14	MLSVRLRLVSNMPRIMLLRAVI	23	983158	983089
29	LTMILPCDSIAPFATPVVPPVYCK	24	MMALRSFVRSPTNGSAIQLAWNKLTMILPCDSIAPFATPVVPPVYCK	61	825518	825700
30	NYTIAMIYCGNTAKLPYLK	19	MMCLRNYTIAMIYCGNTAKLPYLKLV	27	1458554	1458473
31	NLFLLIMK	8	MMVEVSGLFNNKIPMPLPTIYTVKPLLKNLFLLIMKFLL	40	724110	724229
32	TALARNK	7	MNLISKPEQRTALARNKWRKRETENLSLSKYFGLGLKCTVVPVLRL	67	434990	435190
33	WIVSNLRSLVEQAGLMMR	18	MNRWIVSNLRSLVEQAGLMMR	22	516463	516528
34	MRCTYAILLWVILIKAVSLQK	21	MPLKRSTYATPLFLVILVRLNKSKKLLMRCVLLKAKIRMRCTYAILLW	61	331911	331728
35	MPPPAAEKNIAR	12	MPPPAAEKNIARYSFASRVI	21	1659228	1659290
36	PHMFLMCGFVFLLAIKIK	18	MQKPHMFLMCGFVFLLAIKIKVVVADHIFALIRHSFAKIHYLF	44	2028968	2029099
37	RRSMLPK	7	MQKRRSMLPKIL	13	2022017	2021978
38	MQLNILYLTMITISQKLMCQVAILLLRK	28	MQLNILYLTMITISQKLMCQVAILLLRKMRRLMIKSNKCAYLPLNRF	48	503708	503851
39	CCCGCICFSCVSVIALTNTCEPLCFK	26	MQPSSTRKVRCCCGCICFSCVSVIALTNTCEPLCFKRSANKARLY	46	1354808	1354945
40	PMQQGLNTLKLILGRTLVK	19	MQVKDKKPMQQGLNTLKLILGRTLVKVLLVN	32	838881	838785
41	LVLMSAMK	8	MRGKLVLMSAMKSK	15	2219181	2219136
42	LRPLPNSENNDNTYCKCK	18	MRKLRPLPNSENNDNTYCKCKIEG	25	2230403	2230328
43	ELTFAFLWLTQAPPMLNVVCLIAR	24	MRLKRIFAVCLISKKKELTFAFLWLTQAPPMLNVVCLIARPMQ	44	1759407	1759538
44	DYTYELHTK	9	MRLVRNKMNLGVFMKLNKTALIATLATLSLSAISAQATIMEASSKGEL	131	334077	334469
45	TLDVIFVNTSK	11	MRLVRNKMNLGVFMKLNKTALIATLATLSLSAISAQATIMEASSKGEL	131	334077	334469
46	AINRDYTYELHTK	13	MRLVRNKMNLGVFMKLNKTALIATLATLSLSAISAQATIMEASSKGEL	131	334077	334469
47	AINRDYTYELHTKGNK	16	MRLVRNKMNLGVFMKLNKTALIATLATLSLSAISAQATIMEASSKGEL	131	334077	334469
48	DSFAIINQVDEMIPLEIVK	19	MRLVRNKMNLGVFMKLNKTALIATLATLSLSAISAQATIMEASSKGEL	131	334077	334469
49	TLDVIFVNTSKDSFAIINQVDEMIPLEIVK	30	MRLVRNKMNLGVFMKLNKTALIATLATLSLSAISAQATIMEASSKGEL	131	334077	334469
50	NDQANPTNNPCNR	13	MRNDQANPTNNPCNRHRS	19	1503597	1503540
51	MSFTTNGAANAVVEPNNK	18	MSFTTNGAANAVVEPNNKLAIKSFFIVNVIL	32	2232585	2232489
52	RAICGMIKPTQPIIPVIDTAAEVMSVADK	29	MSKLKKKGAPTREVIMPKGTSMEKIERAMVSVSSKKVAPINKLVGN	152	1503773	1503317
53	SMVKVIVSVVNWNK	14	MSLFVVFANILKIIQKPQKLSPRSMVKVIVSVVNWNKLDNSKSL	45	243076	242941
54	PIVMAILSLLSHSIGK	16	MSLQRILCVIPHGHASCYSSKKNWHHYLGKPIVMAILSLLSHSIGKAL	52	241063	240907
55	ILPIGAKVAVWGGGHFGVLNHAYAMCCNK	29	MSQKILPIGAKVAVWGGGHFGVLNHAYAMCCNKS	35	826506	826401
56	SIGLTLCGMVEDPISPAMVRCLK	23	MTALAKPRFPSEFSKSIGLTLCGMVEDPISPAMVRCLK	39	2092508	2092391
57	IKNNGSRIGSCALCHHR	17	MTCGSRLHRIKNNGSRIGSCALCHHRNTVTFTPHLQLLNRRRTERIT	50	1718868	1718718
58	KMRISK	6	MTGTACAIKRSATASVQEQKKMRISKPLV	30	1538874	1538784
59	GRGNQNQPK	9	MTKLKGRGNQNQPKRNRDQQKVFFCF	27	537587	537667
60	EMSNLSASSASIFR	14	MTKVQRFWYACTTKPIPLKCAKSGSANKRSTKSKEMSNLSASSASI	78	679913	679679
61	LKGISSLHPLFHVLIK	16	MTSMLWQKLKGISSLHPLFHVLIKYV	27	832047	832127
62	KPSSLVSAILR	11	MTVPTRVKEIAIPKARVNKPTFRFAMAEEATIGRIGKTHGERVVSNP	99	1914086	1913789
63	MVLISIVVVR	10	MVLISIVVVRQKR	14	2122063	2122104
64	TMYTVSVVLGVRGR	14	MWLHVVYISLMCRMCLIMICQMIARTMYTVSVVLGVRGRVECRLVL	53	2161454	2161612
65	LSGMAVPSSFTKIGTTFFPTKR	22	MYFTISKLSGMAVPSSFTKIGTTFFPTKRIGVSSSSFNRTCFLKGMF	59	595885	595708
66	VICCVQNHEKIAQTLLHLVLSRMK	24	MAAGLRAGKSRTIGLIVPDFENASFARIATHLEAGFRQKGYQLVIAC	277	1021842	1021011
67	SSCCCAEISSRALISGRTFNK	21	MACKIFSVRRKNKYAWRRSSCCCAEISSRALISGRTFNKAGCCRAL	94	442743	443024
68	PAYCVCKPPTMARTSPK	17	MAGFTKEPIWRLALKARLKPAYCVCKPPTMARTSPKR	38	531350	531463
69	PIVSQYNKLYQLIWER	16	MAITTGQNWKKPIVSQYNKLYQLIWERYSLPFCVFKTLNKNLWLSQ	47	2078221	2078361
70	HMHHTVELIDTPAVR	15	MAKTIKVTQVRSSIARLPKHKATLRGLGLRHMHHTVELIDTPAVRGM	60	1589941	1589761
71	GIKVHSITKPPK	12	MAKWSRKCVTHIAVAPQTATGITTKFTKGIKVHSITKPPKLKLSALFS	66	1902434	1902631
72	SCLKPFGLKNAFWK	14	MALIGTPFNLLYNTINAEKESAEAQPSASKQLKIYTCGMGKVGCAKL	82	379666	379420

73	KINFLLVMNILSEDLKVELLQATVVR	26	MALIVCIRKINFLLVMNILSEDLKVELLQATVVRIFHKPLASLFIHKKLT	81	63767	64009
74	LVSEILCLRPVMPMLPLLASAPRAR	25	MALVPSVLFPHLYALLRLKQQSSLNEKLVSEILCLRPVMPMLPLLAS	71	1466089	1465876
75	ATGIIKAPPK	10	MASRATGIIKAPPKPCNTRARTRVSSVLEMAQATDPITKMLTAMRKI	70	2010039	2010248
76	SNACNSMITPIKEIGIVITGMRIAR	25	MASSTTSPIAKIKASKVIKLMEKSNACNSMITPIKEIGIVITGMRIARNE	84	1331508	1331759
77	AASIILPTMILASDGFSNKYCCK	23	MAWFLRLRPCFAEPPAESPSTRYNSDNAGSRSWQSASLPGKPPKS	142	1669694	1670119
78	CITNVLMNPLITNNRTLMRAK	21	MAYHQITLLLPKCITNVLMNPLITNNRTLMRAKSHVN*	38	927221	927334
79	MCFSDKSCMASSANNPSLLSCR	22	MCFSDKSCMASSANNPSLLSCR	23	2043270	2043201
80	SKMELYPEYRVQVLPTGK	18	MCGESFHSKALTCFFRSKMELYPEYRVQVLPTGKLHNHWRQLDFY	343	1795331	1794302
81	MDEKICYSNAVIFYYFNTEKIMAR	24	MDEKICYSNAVIFYYFNTEKIMARRPLVMGNWKLNGSKAFTKALIED	283	1506266	1505417
82	EFIMQNPK	8	MDELFRTRELKFKEFIMQNPKDDVLYAPIEWQDHSEGYTDILYHKSL	302	1291436	1290530
83	PSTFCNVGVTSATSKPKIR	19	MDFILLFAPIFLFAIAAMTRTKIKIGATARNERTKMVPNTPTFSAKLKP	102	1046941	1047246
84	MSSVILR	7	MDFVATILKMNLMCRLVLIKDITFQKMSSVILRKKHKMQSLFVRISNK	68	1414250	1414046
85	SFCRYFLTLK	10	MDKYLQLFYIMWIFLRHFHPLNSEPHPHKYQDPRFSIPVHQKSFCRY	59	1517503	1517326
86	HQMTLNLSDEIHNYYDNNQKR	21	MDRLLSETFKVCFLQRRFIFRRHQMTLNLSDEIHNYYDNNQKRGST	55	1587981	1588145
87	SSKLLMSCHHHAFVQSSTVTDLFSR	25	MDYRLSLHLHGYKEYGGLMAKQMPNLAYFLSSMDQRYHGLPFGYL	79	476948	477184
88	CTNKSPSSTWAIGPSVSAMNSSSFSPSPGCK	31	MEIDPRSSASKCTNKSPSSTWAIGPSVSAMNSSSFSPSPGCKVI	45	301595	301729
89	IRFTDACDNILNTLTFGIDGGIK	23	MELVIHCQIKRDIHGFNKHITTIWIATKIRFTDACDNILNTLTFGIDGGI	52	208617	208772
90	IHKILQNFCTANHK	14	MFAFWNKIHKILQNFCTANHKPINSCTLTNTDLLCIIVGSRYTLVTFE	71	2043490	2043277
91	HTKCHTNGEQNCHVVNNGGTRLDQEGSK	28	MFIFRHTKCHTNGEQNCHVVNNGGTRLDQEGSKLAFSTPTIRVNPV	51	1030690	1030537
92	RVWLILFIMSISLCYFMLSLTNVINIR	27	MFLKRKIRGLGVETAANKGLGNKDAPNTIPPKDNHLRRVWLILFIMS	79	308225	307988
93	SHFCNISLTTWRCK	14	MFSNICPRCRSLKLNKCVSHRCDGTIFCIALNIVSIRPGYWRSHFCN	59	703368	703544
94	MFSQGIKTLSNTKMASFSSK	20	MFSQGIKTLSNTKMASFSSKRVESGKSNGEPVAPAASS	39	1722251	1722367
95	KAFALMLLILAINMILK	17	MFTFFLICLAVGAFAGFLAGLFGIGGGLVIVPVLVYLMPKYVGVPEA	265	109059	108264
96	YNKFNAIGQIQKSR	14	MFVTHWLRYNKFNAIGQIQKSRGSSAKQRRSYWLVFLMLN	41	1498090	1497967
97	NMSVHQINTMIDNK	14	MFYSMDFLVKYRVFLLHKNMSVHQINTMIDNKLLIPLLE	40	664759	664639
98	GQLCHCLGERSGECARR	17	MGICCRRTQTRLLLTRGQLCHCLGERSGECARRLISTQHLKGMAM	217	1821267	1821917
99	LISTQHLKGMAMQRWSK	17	MGICCRRTQTRLLLTRGQLCHCLGERSGECARRLISTQHLKGMAM	217	1821267	1821917
100	SLRAINAALSCELSNCPR	18	MGKDSASACNAVTAPLISSGVSPRSLRAINAALSCELSNCPRTIAVK	64	670372	670563
101	EAGEKAKIELSSAQQTDVNLPYITADATGPK	31	MGKIIGIDLGTTNSCVAVMDGDKPRVIENAEGDRTTPSIIAYTQDNET	635	866033	867937
102	NCWMCMPNAKLKK	13	MHGYVHVKKPRKKFVMWRRNCWMCMPNAKLKKALLSIMIVKRFN	56	1226828	1226660
103	QRLRQQNQR	9	MHTKALYHEGRWCLLLMRFLLRQRLRQQNQRVLALVQLLSWLNEY	55	1309736	1309900
104	FAIGLMTQVECLGWMSESK	19	MIANDNDGMIIFILHLKFAIGLMTQVECLGWMSESKVCFIADI	44	1622803	1622671
105	RHAIIAITELICDVNELAR	19	MIKKIAVLTSGGDAPGMNAAIRGVVRSALAAGLEVYGIYEGYYGLYH	322	95079	96044
106	ENHIINK	7	MIKRRENHIINKILIVSLEMIKCIILLKKDPWREENCFYLVS	43	1370590	1370718
107	NHLSAISK	8	MILKKNHLSAISKCRVFFIFIKCDS	26	1014771	1014693
108	KFQPNSKGK	9	MILQSKKFQPNSKGKRYYRSRVKNNKMTEKGF	33	696643	696741
109	RKCCLSPFIITINVYVLINR	20	MIMLSGKIMRRKCCLSPFIITINVYVLINRLPMWN	36	2212744	2212851
110	VLVMVLMSIIFSSTSCAMSLSPVLIK	26	MINSGGRPYFSSTPSRSNKVLVMVLMSIIFSSTSCAMSLSPVLIKTFF	87	1254672	1254411
111	RAILQRLK	8	MIRRAILQRLKRNNKSRLVRKKKPA	26	418801	418878
112	PPPIITTSKFIVIR	14	MITHCLPCVCNAMPHVKPARPPPIITTSKFIVIRPLLCLLFKRFYII	48	1627509	1627652
113	CLCCLWIMHFQMKSCMLLSNVFK	23	MKCLCCLWIMHFQMKSCMLLSNVFKTVWFFPLNYLNFAANRN	43	1925717	1925588
114	VKVATKPLWHK	11	MKFDIFIHRESRVKVATKPLWHKSNAFE	29	2223339	2223425
115	EFLISLTTAQNNSSR	15	MKIREFLISLTTAQNNSSRLLL	23	530354	530285
116	PFLNLLNK	8	MKKNVLVIGAGGVSQVVVHKCAQHNDVLGKISIASRKIEKCRAIADS	413	437115	438353
117	NVGLLSPLPMVQVNYRIILNLCLK	24	MKNVGLLSPLPMVQVNYRIILNLCLKN	28	1676813	1676896
118	VSVMQVMMNLSLHLR	15	MKQKVSVMQVMMNLSLHLRLKSQLTHS	28	1545379	1545462
119	GGKLTGCWVCKNCK	14	MKRRGGKLTGCWVCKNCKNCVCK	24	1135373	1135444
120	LIMLYLSHGLGMIVLIIK	18	MKRVMWKLIMLYLSHGLGMIVLIIKY	27	1325474	1325393
121	IIQLNKYDK	9	MKSVYFFLPFVCASFSLNAANTSIPMLDKSYQESSQQQEEPIFTCRT	131	1696586	1696193
122	LISESHERPLSLVESVGVR	19	MKYCFKVTKLISESHERPLSLVESVGVRLHRLMCLRCDNFGKNCES	67	235156	235356
123	STASTSASGKRSAQAIAIQPLPVHMSK	27	MLICATSSASEDRSTASTSASGKRSAQAIAIQPLPVHMSKIRLGSCG	55	460222	460386
124	RLPPVAHWVCQLMTCK	16	MLKEIIIVRRVINLHFYSHSHHAQVHCVRFVVLRLMSARCKSRTGRH	91	1243337	1243609
125	VLYEMDGVSEEIAR	14	MLQPKRTKFRKVHKGRNRGIAAGTEVSFGTFGLKAVGRGRLTARQ	137	1594475	1594064
126	RCMIWGLPMLNVK	13	MLRKCKRCMIWGLPMLNVKWRHLRHF	27	740735	740815
127	IACLNDIYFAAWNACSK	17	MLSPVLFKQAGKIACLNDIYFAAWNACSKSAKISWILSIPQERRIKPA	61	869858	869675
128	SLSLNSALSSTDGSDNWCLPIWILSSNLPR	30	MLSTSSDELEWLSSISRFLRGSSSRSSIGDNFFFSNWILLSINKFGIS	121	77923	77560
129	QFRSFCNDISRHDICINNR	19	MLTKIIDYFGKQFRSFCNDISRHDICINNRYAKCHE	37	114906	114795
130	DNGPKKVTK	9	MMARHQELAGFTRDNGPKKVTKWQTVITLIHRT	34	477136	477034
131	ILVYYLCIIGHNVVSVIYLKK	21	MMKILVYYLCIIGHNVVSVIYLKKPPFFVV	31	197179	197086
132	RWIISIPTIIYCCSIR	16	MMKRWIISIPTIIYCCSIRYAKILLV	27	14491	14410
133	SYYMSGDFSLADTIR	15	MMKSYYMSGDFSLADTIRKQV	22	1006747	1006681
134	PAIANSNGPFSCHSVSGVYSVNQSTRIPIKR	31	MNAHTILNNSKPAIANSNGPFSCHSVSGVYSVNQSTRIPIKRKSTPS	88	1496730	1496993
135	TVMSEMGQAMIGFGSCKGTAGEGRAEEATR	30	MNMFEPVDYDLGEMDGALIKVVGVGGGGGNAVNHMVANMIKNDIG	437	173772	175082
136	INGDKMQCGQIFTCMLIYHWIICTTLHR	28	MNRLKVKRIIHVQRRFRINGDKMQCGQIFTCMLIYHWIICTTLHRITR	54	204780	204941
137	CPLFAVKLANTNSLSMPSAVCK	22	MPCVVRRFSPIPLIMAFRIVSMLKCPLFAVKLANTNSLSMPSAVCKC	79	1995957	1995720
138	PISTTINATMPNQMVLSCADSPQKSK	26	MPGTQAITAIRIRSEAINGNTHFDVANNGVSLSILLIINKFMPTGGVIK	81	1727311	1727068
139	WVNMPICTFPHNISKNWIMALTFMRHK	27	MPHHNLSVNSNSLILITSRYLCGNALICSSFKNKICFFGPLKAKHKKP	105	2089154	2088839
140	HLGSILYRMSGQYIFVIKIAHNVLLMVK	28	MPHWKTVFTNFSKNSPHLVSILKKHLGSILYRMSGQYIFVIKIAHNVL	64	797096	797287
141	ALANAIPAMVDALCIFSRASGSLSFTER	28	MPKKALANAIPAMVDALCIFSRASGSLSFTERSKCSNTSLIACRAKP	58	1642718	1642544
142	NLKSVLMLK	9	MPKLTNLKLIFLRWRNLKSVLMLKSPPAQNACS	34	2033703	2033601
143	HLSTQDNLVYVECLSIR	17	MPLAHRLFPTPEHIPFPLLPHALPQARLNYPLTLVQSPHLSHQTPHK	80	2100283	2100043
144	GSIAASSINNHLSANAWGSCAICSQSKSGDLK	32	MPLKVDVSSSNKLKGSIAASSINNHLSANAWGSCAICSQSKSGDLK	119	1231711	1232067
145	HHVPFQFFDAVSPSSQLDFLIQR	23	MPMTNYVISLSSARERRQHVMNEFSKHHVPFQFFDAVSPSSQLDF	281	1342383	1341540

146	QRQFSAIAGFFSPDDR	16	MPRLFPHTNLVISSSDVFLAPHYAHIVHRRQRQFSAIAGFFSPDDRH	60	2110865	2110685
147	NCVRITRNR	9	MPSSRASTAAGNNPPRLTAIMPFHCEAGSKRSDNLRACIWNMSQV	100	1560776	1560476
148	FSTNSTK	7	MQDAPDDNRTKFSTNSTKYSARGPTSQAPRKCGQITRCFKNQTR	45	137103	136968
149	KGFTGICSWKCTIK	14	MQKLRKGFTGICSWKCTIKLVFI	24	1949901	1949972
150	CRYVGQNVAVIVLMSLVIK	19	MQKNPWKCRYVGQNVAVIVLMSLVIKMHYLVSFKVAFTKNYVKCRL	49	256629	256482
151	KTLLMQSLK	9	MQKRRKTLLMQSLKLAKNNFV	22	1546525	1546590
152	SAQPRLSRFHTCSGNSSASSLPNISFIR	28	MQSKKKSAQPRLSRFHTCSGNSSASSLPNISFIRFLLKPYWLTSKTK	52	383388	383543
153	CFSTRPPISNCHLPNSTCSIK	21	MRCFSTRPPISNCHLPNSTCSIKKRLC	28	1273204	1273287
154	IFGLFSVKLSLIFSNVDNLCPCMLLFNR	28	MRIFGLFSVKLSLIFSNVDNLCPCMLLFNRRTSLVNNASRFSF	44	1655705	1655836
155	QSTFARSELSRGTR	14	MRISILKGSHYGKNQKQSTFARSELSRGTRKI	33	2194125	2194026
156	PHWMISLVEEMLVVALQREDQIR	23	MRKLHNAKRIKPHWMISLVEEMLVVALQREDQIRIVREAKEAVQHW	61	1141251	1141068
157	ERIKLTGELPSPLSPPK	17	MRKVCQVVQNLQCHNKKENNMQELERQASLLDAVNLKKYYPVKK	347	270567	271607
158	PRSASSNWGEETPK	14	MRLANRSSPKVFNTQAKSCSDTCCSHSLADKPCVVSIRISSGPSFIK	92	159005	158729
159	VMKIQDCIVKMASMMPFLCGWFISVFR	27	MRLIRVMKIQDCIVKMASMMPFLCGWFISVFR	33	520318	520416
160	LICPAFDQFWRTISSK	16	MRLYNRLICPAFDQFWRTISSKNNQIYSRLTRFNNSRI	39	59976	59859
161	NKFTRNLSIFITIFTWLIGK	20	MRNKFTRNLSIFITIFTWLIGKNILHNNLPIFHSHQFRNIHDFP	45	1000162	1000296
162	MTAPASTSSFTKWTEVPCSRSPASR	25	MRNSCALTRCSMSSIRSSAKIGTFACKMTAPASTSSFTKWTEVPCS	55	16878	17042
163	NVSPMPSAKSMPNPTALFTAPAR	23	MRNVSPMPSAKSMPNPTALFTAPARKPPASVMPKCKGCLMAFANK	47	2252441	2252300
164	RHFHNR	6	MRRHFHNR	9	1119543	1119569
165	AALSRLVVIPMAVLLAR	17	MRRHKNGRKSNARFRAALSRLVVIPMAVLLARKPYPIKVMVIK	44	422255	422386
166	MNSSPK	6	MRSFLVTILLCNPVMGKMKMNSSPKQKDQSPKWMMAIY	39	301748	301631
167	LRKPSSPSTSK	11	MRSSRLRKPSSPSTSKIQLISAPARCSISLSESTKSILNSCANWRPIV	74	1416123	1416344
168	PVQLKCGIILPQIVARFR	18	MRVINSHFLLSIHRLSWRISPLRHLIIDLWQSFLFHQSDDIYIQFHLSR	114	1561118	1561459
169	TDSFNLPCLRATTACAHFNK	20	MRVSKGAGKFSERSWLNCAFAASFSRFCVKAIASCKRAGCKLGFS	82	1714085	1714330
170	LDVGSTEEHCANLSLISQK	19	MRYAILLIPIFLLISCFDKLPFLKANNASQASHDTIAIQSDFYILAQRFQ	262	1152608	1151822
171	VSLLNVYLQR	10	MSATCHTDLIPLNLLIRLEDLQLPFHKQHPFQPKQSLLGGNKYLFYL	70	598262	598052
172	MSGVLRNLMCCWCFQAQR	18	MSGVLRNLMCCWCFQAQRNIMTKILD	27	370605	370524
173	MSKMDYANHPIFDCMPSKISTISHLVITK	29	MSKMDYANHPIFDCMPSKISTISHLVITKVICSRGGTQIRKPIFKSVC	53	31358	31516
174	FGVSAAAAAVAVAAGPAEAAEEK	23	MSLTNEQIIEAIASKSVTEIVELIAAMEEKFGVSAAAAAVAVAAGPAE	123	1961263	1960894
175	LMTCKKSGSCGLPFCIFSTK	20	MSNKRLWYLCNTLGTTASCSSSGSLLKPSTCKNSSRKLMTCKKSG	97	125055	125345
176	KAMIMPPISTVVKR	14	MSPLVERRKAMIMPPISTVVKRSTALVDNQTNPASTQQKSKDPKPR	59	1386806	1386629
177	TIHNAVEVK	9	MSRVAKAPVSIPAGVEVKLDGQLLTVKGKNGELSRTIHNAVEVKQD	178	1591364	1590830
178	NYFVALSLNFLFLCHMLDK	19	MSSHLNKLTLHGLSDSLISSDTWGNRNYFVALSLNFLFLCHMLDKA	81	1344534	1344776
179	TRMQAKQLYVVANVVLMPILFCILAFHFK	29	MSVMTCSKRTRMQAKQLYVVANVVLMPILFCILAFHFKGGEYATRN	54	184203	184041
180	AVINIAHSVSCLIRVVK	17	MSVSQKLKNSIVYQNRVQKVQLLSYPLWKAVINIAHSVSCLIRVVKK	70	1180787	1180577
181	NAIRLALMALTAAIALR	17	MSYEQTTLIANPPCEVSRYLAFISLPVSYIVSMTLSKVTRGLVVRRNA	143	35838	36266
182	PRLCSMAISAAIHTCSGLPPRISVK	25	MSYEQTTLIANPPCEVSRYLAFISLPVSYIVSMTLSKVTRGLVVRRNA	143	35838	36266
183	VVSLGDVVEVMVLEIDEER	19	MTESFAQLFEESLKELETRQGSIVSGTVVAIQKGFVLVDAGLKSESA	550	945568	943918
184	TLMRMLNVNFNVQCIMHK	18	MTKTLMRMLNVNFNVQCIMHKSTVRQSRLVIHVKIQ	37	1707126	1707236
185	ISTCNPVNHLNLMVGAGSASGILSAK	26	MTMKAPSKPCIGNCLKKLAYRLKMSKYYIFQNIGYVINYQNVCYVTT	108	109537	109213
186	TVKPSINCAKSSIICATALMTVVK	24	MTMRQISANSAPKPGMPMLMKTVHGSIIRTAKAPMIWDVFMAYKG	77	107244	107013
187	DARFSAMASARK	12	MTSATTFECCLSGVKFTTKSALGINSSYVPTLKPFSVARWKDARFS	55	1478352	1478187
188	KLPIYVIELSSLTVVRSQVNCSMNK	25	MTSKKLPIYVIELSSLTVVRSQVNCSMNKSRSPTYWHSLQIPSRASI	59	1444620	1444443
189	MTVRVNAIDAEISIPTQYIAK	21	MTVRVNAIDAEISIPTQYIAKLSGLIEGIPGKPAAPKAMALTLNKAMTA	63	2085652	2085840
190	AMALTLNKAMTALEMPAPIMADMK	24	MTVRVNAIDAEISIPTQYIAKLSGLIEGIPGKPAAPKAMALTLNKAMTA	63	2085652	2085840
191	KISASLMTSANVRLPLSSIAYNFLVSSMR	29	MVFTMSWVNVPAAEQPKKISASLMTSANVRLPLSSIAYNFLVSSMR	133	2430	2828
192	MVFVVWKNSPHRLK	14	MVFVVWKNSPHRLK	15	1978431	1978475
193	LTVLKKILAIILMQWDLILR	20	MVGRRLTVLKKILAIILMQWDLILRRNYGKCH	33	1579765	1579666
194	CVNVSLNGSCRIFIISVLMCWCVPPLLKR	29	MVKCVNVSLNGSCRIFIISVLMCWCVPPLLKRGLMSLPQIRSLLSVRI	52	1225946	1225790
195	HKMFPVFHSVYCGVREPLIK	20	MVKIIRHKMFPVFHSVYCGVREPLIKNMIFFAIKH	36	2089498	2089605
196	PPNQATNNPTATASHIFQFRK	21	MVPTNNPVNNTGCAPINPTPRPPNQATNNPTATASHIFQFRKTAKC	49	910562	910708
197	VRLIMMWQGQTTPSVIK	17	MVSLASVSRVRLIMMWQGQTTPSVIKPHYRQPSKPLTVYAIPQART	62	95428	95613
198	NLMITWRTRNPMVTNLSILLSSAK	24	MVVLNTSIAFGAKCRKNILNLAISTMFVQCELSSLNCKIVIPHLALCIP	102	2107394	2107088
199	LVFLGGK	7	MWALKLVFLGGKNIFMPFIKK	22	1087064	1086998
200	YREHTTCHFENVSLAGTNTPSFR	23	MWRNRAIRKYREHTTCHFENVSLAGTNTPSFRQKWEYFLF	41	1192539	1192661
201	TEFDRNLPFTVLSAPQVNEESVAQTVAKIK	30	MYNTLNNKEDISLVSNNFGFLRLPVHFNPVETDAEWVITGVPFDAS	307	1576535	1575614
202	PNSSVIKPLNMACRGVLLLSMVFLSVPIQK	30	MYTWINLIPQKKKKPNSSVIKPLNMACRGVLLLSMVFLSVPIQKITAT	59	2124007	2123830
