# Supplementary material for: AgBase: a functional genomics resource for agriculture
Source: BMC Genomics. 2006 Sep 8;7:229. doi: 10.1186/1471-2164-7-229 (PMC1618847; doi:10.1186/1471-2164-7-229)
Supplement: Additional File 3 — Location of ePST47 in P. multocida Pm70 genome. Region of PM70 genome coding for accA and guaA genes and the 5' end of PMO291 (A). The position in genome is on the left hand side; DNA sequence is shown above; amino acid sequence below; direction of reading frames shown by arrows. The ePST47 is located in the intergenic region between accA and guaA. The figure is taken directly from NCBI nucleotide database graph view with the accession number NC_002663. An expanded view of intergenic region between accA and guaA including ePST47, which runs in the opposite direction to accA, guaA and PMO291 (arrowed) is also shown (B). The overlapping peptides used to identify ePST47 are indicated using bold type (PRIDE accession number pending). This region of the PM70 genome also has 60% identity (* indicates identical nucleotides) and 74% similarity at the protein level with the Haemophilus ducreyi hypothetical protein HD_1218 (Genbank accession AAP96060). [file 1471-2164-7-229-S3.pdf]

A.

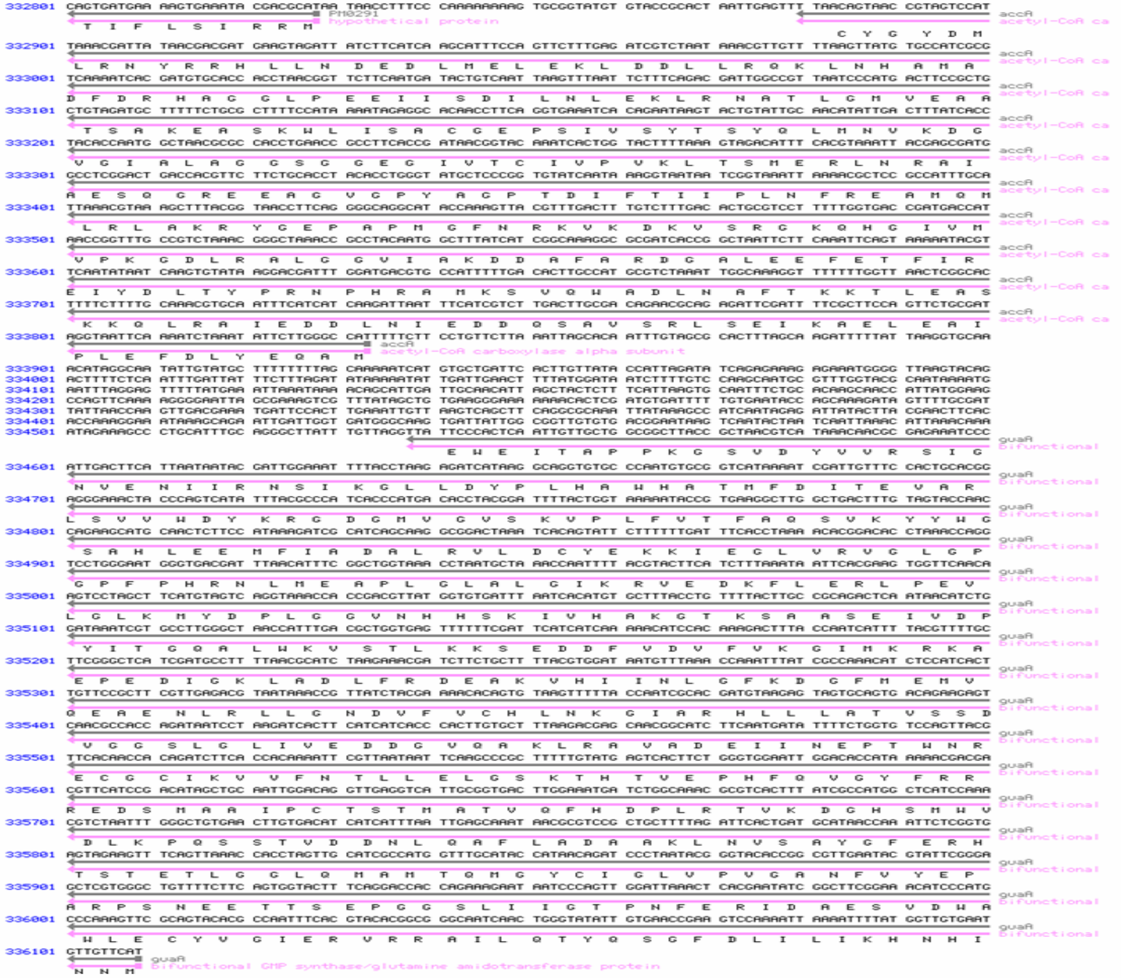

B.

```
Pm70      TTTCTTTAGATATAAAAATATTGATTGAACTTTTATGGATAATCTTTTGTCCAAGCAATG 334079
ePST47    ---------------------------------------------------------ATG 3
HD_1218   -----------------------------------------------------ATTCTAG 7
                                                                *     *

Pm70      CGTTTGGTACGCAATAAAATGAATTTAGGAGTTTTTATGAAATTAAATAAAACAGCATTG 334139
ePST47    CGTTTGGTACGCAATAAAATGAATTTAGGAGTTTTTATGAAATTAAATAAAACAGCATTG 63
HD_1218   TGTGCATTCTTCAATGA--TTGGTTTATCGTTGGCTAGGAGTGTTGCTGTGTTTCCTTTT 65
           **     *    **** *  *    ****     *    ** **    *    *         * **

Pm70      ATTGCAACAT--TAGCTACTCTTTCATTAAGTGCAATTTCTGCACAAGCAACCATTATGG 334197
ePST47    ATTGCAACAT--TAGCTACTCTTTCATTAAGTGCAATTTCTGCACAAGCAACCATTATGG 121
HD_1218   GTGGCTAATTCATAGTCGTAATTCGGGTTAATG-GGTTTATACACC-GCACCTGAGGCAG 123
           * ** *  *   ***         **     * * **    *** * ***  *** *        *

Pm70      AAGCCA-GTTCAAAAGGGGAATTAGCGAAAGTCGTTTATAGCTGTGAAGGGAAAAAAACA 334255
                                                                    T
                                                                    T
ePST47    AAGCCA-GTTCAAAAGGGGAATTAGCGAAAGTCGTTTATAGCTGTGAAGGGAAAAAAACA 180
HD_1218   ATTTTACATTTTTCATTGGGATCATTTCATCTACTTGATTG--ATGATCGCATAAGAATC 181
          *    *  **    *  ** ** *      *   *  ** ** *     ***   * *  * ** **

Pm70      CTCGATGTGATTTTTGTGAATACCAGCAAAGATAGTTTTGCGATTATTAACCAAGTTGAC 334315
                                          D  S  F  A  I  I  N  Q  V  D
            L  D  V  I  F  V  N  T  S  K
            L  D  V  I  F  V  N  T  S  K  D  S  F  A  I  I  N  Q  V  D
ePST47    CTCGATGTGATTTTTGTGAATACCAGCAAAGATAGTTTTGCGATTATTAACCAAGTTGAC 240
HD_1218   TTTATCGGTATTCACGAAAATAACATCGAGTGTATTTTTATCTTTA-----CAAGCATAT 236
           *    *  ***   *   **** ** * *    ** ****      ***      ****   *

Pm70      GAAATGATTCCACTTGAAATTGTTAAGTCAGCTTCAGGCGCAAATTATAAAGCCATCAAT 334375
            E  M  I  P  L  E  I  V  K
                                                            A  I  N
            E  M  I  P  L  E  I  V  K                       A  I  N
ePST47    GAAATGATTCCACTTGAAATTGTTAAGTCAGCTTCAGGCGCAAATTATAAAGCCATCAAT 300
HD_1218   ACAAC--TTTGG--TGATTTCATTCTCTGAGCTTT-GTTGCA--TTATGGTTGCGTGTGC 289
            **   **     ***  *  **   * *****  *  ***   ****       * *

Pm70      AGAGATTAT-ACTTACGAACTTCACACCAAAGGAAATAAAGCAGAATTGATTGGTGATGG 334434
               D  Y  T  Y  E  L  H  T  K
            R  D  Y  T  Y  E  L  H  T  K
            R  D  Y  T  Y  E  L  H  T  K  G  N  K
ePST47    AGAGATTAT-ACTTACGAACTTCACACCAAAGGAAATAAAGCAGAATTGATTGGTGATGG 359
HD_1218   AGAGATGGTTGCTAACAAGCCT---ATCATTGAGAATAGCGC-GGTTTGTATTTCAT-- 343
          ******  *  ** ** * * * *    * **   *   ****  ** *  ***  *   * **

Pm70      GCAAGTGATTATTGGCGGTTGTGTGACGGAATAAGTCAATACTAATCAATTAAACATTAA 334494
ePST47    GCAAGTGATTATTGGCGGTTGTGTGACGGAATAA-------------------------- 393
HD_1218   ------------------------------------------------------------
```
