# Supplementary material for: AgBase: a functional genomics resource for agriculture
Source: BMC Genomics. 2006 Sep 8;7:229. doi: 10.1186/1471-2164-7-229 (PMC1618847; doi:10.1186/1471-2164-7-229)
Supplement: Additional File 4 — Channel catfish proteins and ESTs identified using the ProtIDer tool. The ProtIDer tool is designed for use with species that have large numbers of ESTs but few proteins in the NRPD. ProtIDer matches ESTs and EST assemblies to highly homologous proteins from other species by TBLASTN-searching the NRPD with all catfish ESTs. For example, the channel catfish genome sequence is not available and there are only 1,108 NRPD entries for this species. When we analyzed channel catfish ovary tissue we were only able to identify 10 proteins from NRPD. Using ProtIDer to create a database of highly homologous proteins resulted in a five-fold increase in the number of proteins identified in this experiment. The proteins identified from the channel catfish NRPD database (cfNRPD), highly homologous database (hpDB) and EST databases are shown here. The ProtIDer tool is available from AgBase upon request. [file 1471-2164-7-229-S4.pdf]

Channel Catfish Proteins (cfNRPD)

1	gi|31339953|sp|O42197|B2MG_ICTPU Beta-2-microglobulin precursor
2	gi|52219464|gb|AAU29515.1| natural killer cell enhancing factor [Ictalurus punctatus]
3	gi|46577102|sp|Q90YU5|RL23_ICTPU 60S ribosomal protein L23
4	gi|47606688|gb|AAT36327.1| Fas receptor [Ictalurus punctatus]
5	gi|28932676|gb|AAO60426.1| sodium channel 7 [Ictalurus punctatus]
6	gi|15294017|gb|AAK95185.1| 40S ribosomal protein S3a [Ictalurus punctatus]
7	gi|44887609|gb|AAS48084.1| myogenin [Ictalurus punctatus]
8	gi|15294021|gb|AAK95187.1|AF402813_1 40S ribosomal protein S5 [Ictalurus punctatus]
9	gi|47117268|sp|Q90YQ4|RS19_ICTPU 40S ribosomal protein S19
10	gi|44887605|gb|AAS48082.1| follistatin [Ictalurus punctatus]

Highly homologous proteins (hpDB)

1	TC6967.p1 [968.43.924 Cerr] similar to ANX1_HUMAN (P04083) Annexin A1 (Annexin I) (Lipocortin I)
2	TC6931.p1 [881.67.880 Cmis] similar to ACTC_FUGRU (P53480) Actin, alpha cardiac
3	TC8258.p1 [2440.107.1231] similar to ACTB_CYPCA (P83750) Actin, cytoplasmic 1 (Beta-actin)
4	CK420691.p1 [858.109.642 unknown] hypothetical protein (EST-Analyzer)
5	TC8481.p1 [1306.99.677] similar to RHOA_RAT (P61589) Transforming protein RhoA
6	CK419489.p1 [619.40.306 Nerr] similar to IDHP_PIG (P33198) Isocitrate dehydrogenase [NADP], mitochondrial
7	TC9232.p1 [1199.157.1199 Cmis] similar to CN37_HUMAN (P09543) 2',3'-cyclic-nucleotide 3'-phosphodiester
8	TC6714.p1 [1226.118.465] similar to B2MG_ICTPU (O42197) Beta-2-microglobulin precursor
9	TC6915.p1 [1074.39.635] similar to TDX_CYNPY (Q90384) Thioredoxin peroxidase
10	TC6799.p1 [514.41.460] similar to RL23_RAT (P62832) 60S ribosomal protein L23
11	CK414619.p1 [858.246.821] similar to RAC1_RAT (Q6RUV5) Ras-related C3 botulinum toxin substrate 1
12	TC9389.p1 [1375.102.1035] similar to RSF2_HUMAN (P50749) Ras association domain family 2
13	BM438442.p1 [783.1.381 Nmis] similar to CFAB_PANTR (Q864W0) Complement factor B precursor
14	TC6961.p1 [1033.49.1032 Cmis] similar to IF2G_HUMAN (P41091) Eukaryotic translation initiation factor
15	TC6897.p1 [972.88.540] similar to NPC2_BRARE (Q9DGJ3) Epididymal secretory protein E1 precursor
16	BE469146.p1 [849.225.847 Cmis] similar to ITB0_XENLA (P12607) Integrin beta-1* precursor
17	TC9768.p1 [723.3.509 Nmis] similar to T134_MOUSE (Q9D7W4) Tetraspan protein SB134
18	CB937074.p1 [841.165.839] similar to ARDH_MOUSE (Q9QY36) N-terminal acetyltransferase complex ARD1
19	BE469854.p1 [628.2.190 Nmis] similar to ACTB_STRPU (P53473) Actin, cytoskeletal
20	BE469527.p1 [776.2.634 Nmis Cerr] similar to HGFA_MOUSE (Q9R098) Hepatocyte growth factor activator
21	NP625806.p1 [1466.1.1464 NCmis] similar to CIN2_RAT (P04775) Sodium channel protein type II alpha
22	TC6693.p1 [1676.1.1485 Nmis] similar to CHIA_HUMAN (Q9BZP6) Acidic mammalian chitinase precursor
23	CB938994.p1 [782.171.665] similar to TPC6_HUMAN (Q86SZ2) Trafficking protein particle complex subunit
24	TC8288.p1 [1842.123.1128] similar to G3P_ONCMY (O42259) Glyceraldehyde-3-phosphate dehydrogenase
25	TC6754.p1 [948.1.855 Nmis] similar to RS3A_ORYLA (O73813) 40S ribosomal protein S3a
26	TC8719.p1 [1187.157.836] similar to GTA_PLEPL (P30568) Glutathione S-transferase A (EC 2.5.1.18)
27	TC7671.p1 [904.3.843 Nmis] similar to LIPL_CHICK (P11602) Lipoprotein lipase precursor
28	TC8702.p1 [1020.81.488] similar to H33_HUMAN (P06351) Histone H3.3 (H3.A) (H3.B) (H3.3Q)
29	TC8601.p1 [1477.58.1069] similar to ANX2_HUMAN (P07355) Annexin A2 (Annexin II) (Lipocortin II)
30	CK418739.p1 [917.2.835 Nmis] similar to IMA1_XENLA (P52170) Importin alpha-1 subunit
31	TC9242.p1 [1446.129.1331] similar to RT29_HUMAN (P51398) Mitochondrial 28S ribosomal protein S29
32	TC8398.p1 [988.1.647 Nmis] similar to RS5_HUMAN (P46782) 40S ribosomal protein S5
33	BE469200.p1 [997.84.911 Cerr] similar to SUH_XENLA (Q91880) Suppressor of hairless protein homolog
34	TC8415.p1 [628.47.487] similar to RS19_ICTPU (Q90YQ4) 40S ribosomal protein S19
35	TC9648.p1 [940.3.744 Nmis] similar to ABB2_HUMAN (Q92870) Amyloid beta A4 precursor protein-binding
36	TC7105.p1 [1744.133.1287] similar to RIR2_BRARE (P79733) Ribonucleoside-diphosphate reductase M2 channel
37	TC7974.p1 [991.53.988] similar to FSA_BRARE (Q9YHV4) Follistatin precursor (FS)
38	TC7181.p1 [1024.113.880 Cerr] similar to IF2A_RAT (P68101) Eukaryotic translation initiation factor
39	BM494897.p1 [429.47.427 Cmis] similar to PDA6_MESAU (P38660) Protein disulfide-isomerase A6 precurs

40 TC9327.p1 [597.54.596 Cmis] similar to FABG_THEMA (Q9X248) 3-oxoacyl-[acyl-carrier-protein] reductase
41 CK419980.p1 [942.177.917 Nerr] similar to ITB3_HUMAN (P05106) Integrin beta-3 precursor
42 TC6628.p1 [1147.99.590] similar to CYPH_HEMPU (P91791) Peptidyl-prolyl cis-trans isomerase
43 TC8746.p1 [877.106.741] similar to GS27_MOUSE (O35166) 27 kDa Golgi SNARE protein
44 BM495435.p1 [501.2.499 NCmis] similar to HA1F_CHICK (P15979) Class I histocompatibility antigen, F1
45 CB940762.p1 [705.2.703 Nmis] similar to ELM1_MOUSE (Q8BPU7) Engulfment and cell motility protein 1
46 CK424308.p1 [937.3.916 Nmis Cerr] similar to SMY1_HUMAN (Q8NB12) SET and MYND domain containing protei
47 TC8362.p1 [1653.103.1233] similar to ACTA_RAT (P62738) Actin, aortic smooth muscle (Alpha-actin 2)
48 TC7279.p1 [1244.469.1175 NCerr] similar to YY46_ANASP (Q8YRI1) Hypothetical WD-repeat protein alr34

Untranslated ESTs

1 TC11388 UP|Q6P3K9 (Q6P3K9) Bactin1 protein, complete
2 TC10436 UP|Q5XJK2 (Q5XJK2) Zgc:101846, partial (88%)
3 TC10059 homologue to UP|ATPB_CYPCA (Q9PTY0) ATP synthase beta chain, mitochondrial precursor
4 TC9911 homologue to UP|Q800W9 (Q800W9) Elongation factor 1-alpha, complete
5 CK421703 AUF_IpSpn_61_p04 Spleen cDNA library Ictalurus punctatus cDNA 5- similar to histone H4
6 TC10105 similar to UP|Q9I9D1 (Q9I9D1) Voltage-dependent anion channel, complete
7 TC11389 UP|ACT2_XENLA (P10995) Actin, alpha sarcomeric/cardiac (Alpha 2), complete
8 TC10060 homologue to UP|Q910C4 (Q910C4) Mitochondrial ATP synthase alpha-subunit, complete
9 BM494161 IpCGBr1_13_G06_21 Ictalurus punctatus Brain1 primary library Ictalurus punctatus cDNA clone
10 TC10174 similar to UP|Q804H2 (Q804H2) Annexin 1a, partial (81%)
11 TC11605 GB|AAH04274.2|48257076|BC004274 H2A histone family, member V, isoform 1
12 TC10211 homologue to UP|Q7T334 (Q7T334) Zgc:64133, complete
13 TC11535 homologue to UP|Q7ZU62 (Q7ZU62) Ik:tdsubc_1f2 protein (Fragment), partial (95%)
14 TC10100 similar to PIR|C28456|C28456 histone H1.11R - chicken {Gallus gallus;} , partial (84%)
15 CK416763 AUF_IpInt_53_i17 Intestine cDNA library Ictalurus punctatus cDNA 5- similar to MAD2L1 binding protein
16 CK420763 AUF_IpTrk_27_k07 Trunk kidney cDNA library Ictalurus punctatus cDNA 5- similar to XP_148064
17 TC11393 UP|Q6DHS1 (Q6DHS1) Actin, alpha 2, smooth muscle, aorta, complete
18 TC12761 homologue to UP|Q6PC12 (Q6PC12) Enolase 1, (Alpha), partial (44%)
19 TC9868 UP|Q6NW90 (Q6NW90) Tubulin, beta 5, partial (72%)
20 CK419489 AUF_IpOva_21_c10 Ovary cDNA library Ictalurus punctatus cDNA 5- similar to isocitrate dehydrogenase
21 TC9991 homologue to UP|Q6NYM9 (Q6NYM9) Zgc:76908, complete
22 TC10214 similar to UP|Q5WU20 Hypothetical protein
23 TC9941 similar to UP|Q6PC16 (Q6PC16) NADH dehydrogenase (Ubiquinone) 1 beta subcomplex, 10
24 TC10129 UP|RS28_BRARE (Q6PBK3) 40S ribosomal protein S28, complete
25 TC9922 similar to UP|Q91219 (Q91219) Simple type II keratin K8b (S2), partial (92%)
26 TC10083 similar to UP|Q803D7 (Q803D7) Sb:cb825 protein (Fragment), complete
27 TC11569 UP|Q643S2 (Q643S2) Natural killer cell enhancing factor, complete
28 CB940355 IpCGJx14_12_B09_23 IpCGJx14 Ictalurus punctatus cDNA clone
29 TC11453 UP|Q90YP1 (Q90YP1) 40S ribosomal protein S30, complete
30 TC9992 homologue to UP|Q6P3J5 (Q6P3J5) Eukaryotic translation elongation factor 2, like, complete
31 CK419560 AUF_IpOva_21_f21 Ovary cDNA library Ictalurus punctatus cDNA
32 TC12903 similar to UP|RLA2_RAT (P02401) 60S acidic ribosomal protein P2, partial (89%)
33 TC10096 UP|Q90YU4 (Q90YU4) Ribosomal protein L23a (Fragment), partial (97%)
34 TC11884 homologue to UP|Q803B0 (Q803B0) Hspd1 protein (Heat shock 60 kD protein 1), partial (47%)
35 CK420617 AUF_IpTrk_27_d14 Trunk kidney cDNA library Ictalurus punctatus cDNA
36 TC13096 homologue to UP|Q6IQM2 (Q6IQM2) Zgc:86706, complete
37 CK420691
38 BE574223
39 CK426292
40 CK418481
41 CK420260

42 CV989760
43 CK414118
44 CK414852
45 CK425397
46 TC11353
47 CV989802
48 TC12693 similar to UP|Q6NSM8 (Q6NSM8) Zgc:66101 protein, partial (21%)
49 TC13120 homologue to UP|Q90YD5 (Q90YD5) GTP binding protein Rab1a (Fragment), partial (94%)
50 TC12257 similar to UP|Q6NZM9 (Q6NZM9) Histone deacetylase 4, partial (4%)
51 TC12552 similar to UP|Q7T345 (Q7T345) Zgc:64115, partial (54%)
52 CK414088
53 TC13191 similar to UP|Q7T366 (Q7T366) Membrin, complete
54 CK418100
55 CK412064
56 CK416857
57 CK415924
58 TC11800 weakly similar to UP|Q8JG42 (Q8JG42) Caspase 3, partial (72%)
59 CK415197
60 TC11773 similar to UP|Q8AVU0 (Q8AVU0) MGC52838 protein, partial (55%)
61 TC13851 weakly similar to UP|Q891V8 (Q891V8) Virulence factor mviN, partial (4%)
62 TC10247 similar to UP|Q6PBR9 (Q6PBR9) Tumor necrosis factor superfamily, member 5-induced protein 1
63 CK425448
64 TC13000 similar to UP|NPC2_BRARE (Q9DGJ3) Epididymal secretory protein E1 precursor
65 CK424907
66 CV995186
67 TC9978 similar to PIR|S24401|S24401 tropomyosin 2, skeletal muscle alpha splice form CTm4
68 TC10042 similar to UP|143B_HUMAN (P31946) 14-3-3 protein beta/alpha (Protein kinase C inhibitor protein-1)
69 TC11582 homologue to UP|Q6DHM9 (Q6DHM9) Small GTPase RhoA, complete
70 TC10384 similar to UP|Q6PC77 (Q6PC77) ATP synthase, H+ transporting, mitochondrial F0 complex subunit d
71 TC10104 UP|Q6YNX6 (Q6YNX6) Calmodulin 2, complete
72 TC12887 UP|RS18_ICTPU (Q90YQ5) 40S ribosomal protein S18, complete
73 TC10216 similar to UP|Q804G3 (Q804G3) Annexin 11b (Annexin A11b), partial (28%)
74 TC10353 similar to UP|Q6IHK7 (Q6IHK7) HDC02322, complete
75 TC12988 homologue to UP|Q6PC12 (Q6PC12) Enolase 1, (Alpha), partial (65%)
76 TC13055 similar to UP|Q90306 (Q90306) G-RICH, partial (55%)
77 TC12869 similar to UP|Q8JJB2 (Q8JJB2) Cold inducible RNA-binding protein alpha, partial (69%)
78 TC12547 homologue to UP|Q90YW8 (Q90YW8) Ribosomal protein L3, partial (42%)
79 TC11716 similar to UP|PDX3_BOVIN (P35705) Thioredoxin-dependent peroxide reductase
80 TC10240 homologue to UP|Q6PBW4 (Q6PBW4) Zgc:73214 protein, complete
81 CV996902
82 TC12877 UP|RL19_ICTPU (Q90YU8) 60S ribosomal protein L19, complete
83 TC10055 homologue to GB|AAH49038.1|29294663|BC049038 zgc:73149 protein {Danio rerio;}
84 TC13122 similar to UP|Q6P0S2 (Q6P0S2) Zgc:77898, partial (83%)
85 TC11425 UP|B2MG_ICTPU (O42197) Beta-2-microglobulin precursor, complete
86 TC11438 SP|P05213|TBA2_MOUSE Tubulin alpha-2 chain (Alpha-tubulin 2). {Mus musculus;} , complete
87 CK422925
88 CB936554
89 TC13114 UP|Q90YU2 (Q90YU2) Ribosomal protein L26, complete
90 BE468503
91 TC10062 UP|Q90YT9 (Q90YT9) Ribosomal protein L28, complete
92 CB939493
93 TC11501 similar to UP|Q7ZVA4 (Q7ZVA4) Zgc:56142, partial (91%)

94	CK415460
95	CK411904
96	TC11523 homologue to UP|Q6TL18 (Q6TL18) HSP-90, complete
97	TC12888 UP|Q90YT2 (Q90YT2) Ribosomal protein L36, complete
98	TC10615 UP|Q90WT7 (Q90WT7) BC-10 protein, complete
99	CV995669
100	TC12938 homologue to UP|Q6Y218 (Q6Y218) Ras-related C3 botulinum toxin substrate 2, complete
101	TC12942 GB|AAK95195.1|15294037|AF402821 40S ribosomal protein S13 {Ictalurus punctatus;} , complete
102	CK417659
103	CK415733
104	TC13288
105	TC12892 UP|RS23_ICTPU (Q90YQ1) 40S ribosomal protein S23, complete
106	TC12316 similar to UP|FLNA_MOUSE (Q8BTM8) Filamin A (Alpha-filamin) (Filamin 1)
107	CK412831
108	TC11785 similar to UP|Q68ER0 (Q68ER0) Chchd2-prov protein, partial (81%)
109	TC13213
110	CV990616
111	CK415335
112	TC11490 UP|RS25_ICTPU (Q90YP9) 40S ribosomal protein S25, complete
113	CK414020
114	TC10207
115	CV992009
116	CK424562
117	TC13605
118	CV993102
119	CK416915
120	CK413231
121	CB939967
122	CK416812
123	TC12028 similar to UP|Q90YX4 (Q90YX4) P27-like cyclin-dependent kinase inhibitor, partial (19%)
124	CK414217
125	CK424024
126	CV994486
127	TC9972 homologue to UP|Q7ZVK9 (Q7ZVK9) Zgc:55951 protein, complete
128	CK422723
129	CF262295
130	CK417534
131	CK414678
132	TC10148 homologue to UP|Q8JHI0 (Q8JHI0) Solute carrier family 25 member 5 protein
133	TC11771 homologue to UP|MO1B_HUMAN (Q9H8S9) Mps one binder kinase activator-like 1B
134	TC11735 UP|Q5XJK2 (Q5XJK2) Zgc:101846, partial (95%)
135	TC11863 GB|AAA38496.1|554015|MUSIGHV01B Ig heavy chain precursor {Mus musculus;} , partial (30%)
136	CK412100
137	CF262894
138	TC10106 similar to UP|Q6PH75 (Q6PH75) Cathepsin B, partial (97%)
139	CV996746
140	TC12955 similar to UP|Q6IQU9 (Q6IQU9) Zgc:86599, partial (96%)
141	CK414635
142	BE469301
143	BM494698
144	BE470286
145	CK418935

146	BM494799
147	CK412965
148	CK421032
149	CK423069
150	CK414040
151	TC9917 similar to UP|Q6TNR6 (Q6TNR6) Peptidylprolyl isomerase A, complete
152	CK411538
153	TC11853 similar to UP|Q71SZ2 (Q71SZ2) Cytochrome c oxidase subunit Va, complete
154	CV992715
155	CK415741
156	BM494423
157	CK419518
158	BM495980
159	TC10601 similar to GB|AAC52724.1|1354501|MMU54639 RalGDS-like factor {Mus musculus;} , partial (6%)
160	CK414803
161	TC10176 homologue to UP|Q7SZR4 (Q7SZR4) Phosphoglycerate mutase 1, complete
162	BE470169
163	TC13007 homologue to UP|Q5SPJ6 (Q5SPJ6) Protein tyrosine phosphatase, receptor type, D, partial
164	CF263658
165	CK412701
166	CB940672
167	CK425552
168	CK413831
169	CV987758
170	CK423956
171	CK417670
172	CK415236
173	CK411897
174	CK413651
175	TC12899 GB|AAK95200.1|15294047|AF402826 40S ribosomal protein S17 {Ictalurus punctatus;} , complete
176	TC13522 similar to UP|Q5TB85 (Q5TB85) OTTHUMP00000016079 (Fragment), partial (51%)
177	CK411005
178	CV996460
179	CK420472
180	TC14075 similar to UP|O93447 (O93447) Annexin max4, partial (51%)
181	TC11378 weakly similar to UP|Q62775 (Q62775) SH3 domain binding protein, partial (11%)
182	BM494415
183	TC9880 UP|Q6IQJ2 (Q6IQJ2) Zgc:55461, complete
184	CV995775
185	CK423025
186	TC11377 similar to UP|Q8VI80 (Q8VI80) Fibroblast growth factor 21, partial (10%)
187	CB936723
188	TC11363 similar to GB|AAB41498.1|1805280|HSU83867 alpha II spectrin {Homo sapiens;} , partial (15%)
189	CK418024
190	CK418389
191	TC11560 homologue to UP|Q6P959 (Q6P959) Mitochondrial ATP synthase gamma-subunit, complete
192	TC9858 weakly similar to UP|Q6CEC8 (Q6CEC8) Similarity, partial (3%)
193	CK422385
194	CK415039
195	CV994751
196	CK425597
197	BM424844

198	BM494706
199	CK417216
200	CK417758
201	CV988733
202	CV991573
203	CK414937
204	CV988587
205	BM494124
206	TC10073 similar to UP|Q8JIP8 (Q8JIP8) Warm-temperature-acclimation-related-65 kDa-protein-like-protein
207	CK421805
208	BM029064
209	TC10296 homologue to GB|AAM53975.1|21435808|AF515726 translational eukaryotic inititation factor 4A
210	CV990065
211	CK423313
212	BM027884
213	CB939552
214	BM029527
215	TC13731 similar to UP|SKB1_HUMAN (O14744) Protein arginine N-methyltransferase 5
216	CK419729
217	TC12129 weakly similar to UP|Q672Y9 (Q672Y9) CC chemokine SCYA103, partial (72%)
218	CK413534
219	CK421744
220	TC12175 weakly similar to UP|O93268 (O93268) Aminopeptidase N , partial (22%)
221	CK413486
222	BM494051
223	CV993251
224	TC10306 weakly similar to UP|Q5U3F5 (Q5U3F5) Zgc:103602, partial (20%)
225	TC10499 homologue to UP|Q6TXI9 (Q6TXI9) LRRGT00010, partial (22%)
226	CV990323
227	CK418029
228	BE470192
229	CK419721
230	TC11759 similar to UP|Q6NYW8 (Q6NYW8) Heterogeneous nuclear ribonucleoprotein A0, partial (74%)
231	CK425323
232	BM438934
233	TC13408 similar to UP|Q7T323 (Q7T323) Zinc finger, DHHC domain containing 4, partial (91%)
234	BM494336
235	CV996550
236	CK413278
237	BE469111
238	CV996663
239	CK419079
240	CV991217
241	CK421667
242	BM494958
243	CV990298
244	CV991507
245	TC11908 similar to UP|Q66I80 (Q66I80) Zgc:103587, complete
246	CK412610
247	CK424018
248	TC13201
249	BM029228

250	CK417256
251	TC12972 homologue to UP|Q7ZV13 (Q7ZV13) Zgc:56283, partial (94%)
252	CK418357
253	CK419254
254	CV991262
255	CK416966
256	CK424403
257	CK424853
258	CB939040
259	CK415258
260	CK418488
261	CK417222
262	TC10003 homologue to UP|Q8JHI0 (Q8JHI0) Solute carrier family 25 member 5 protein
263	TC13121 similar to UP|Q8AVH4 (Q8AVH4) MGC53417 protein (NOB1 protein), partial (83%)
264	CK415238
265	CK420747
266	TC10185 UP|MVP_ICTPU (Q9DGM7) Major vault protein (MVP) (Fragment), complete
267	CK419262
268	TC10625 homologue to UP|Q6DGJ6 (Q6DGJ6) Zgc:92891, complete
269	CV996769
270	TC13810 similar to UP|O97757 (O97757) Superfast myosin heavy chain, partial (6%)
271	BM438369
272	TC10041 UP|Q90YR9 (Q90YR9) 40S ribosomal protein S5, complete
273	BE468421
274	TC13491
275	CK425301
276	TC11833 homologue to GB|AAH02602.2|37589900|BC002602 UREB1 protein {Homo sapiens;} , partial (40%)
277	TC11367 similar to UP|Q751I3 (Q751I3) AGL277Wp, partial (3%)
278	CK413326
279	CF261686
280	CK416206
281	CF263757
282	CK417514
283	BM496167
284	CK412150
285	TC11574 similar to UP|Q6P603 (Q6P603) Annexin A2a, complete
286	CV987986
287	CK425028
288	CK421973
289	TC12106 weakly similar to UP|MYOC_BOVIN (Q9XTA3) Myocilin precursor
290	CK420777
291	CV993748
292	CK417601
293	CV989743
294	CV989735
295	TC11533 similar to UP|Q6P6Y0 (Q6P6Y0) Transaldolase 1, complete
296	CK419392
297	TC13481
298	TC11430 homologue to GB|AAK95132.1|15293879|AF401560 ribosomal protein L7a {Ictalurus punctatus;}
299	TC11699 similar to UP|Q86XB5 (Q86XB5) LOC136288 protein (Fragment), partial (14%)
300	BM029046
301	CB938495

302	CF261661
303	CV989006
304	BM438928
305	CK421941
306	CF263388
307	BE213004
308	CK410776
309	CF263546
310	CF262962
311	BM496673
312	TC13622 weakly similar to UP|Q98C58 (Q98C58) Acetyl-coa synthetase, partial (6%)
313	CK414007
314	CK420866
315	CK420274
316	TC9926 UP|Q8CHU4 (Q8CHU4) Pi16 protein (Fragment), partial (4%)
317	BM438759
318	CK425679
319	TC14152
320	TC14102
321	CK423915
322	CK412904
323	BM439119
324	CK414630
325	CK423887
326	CV992447
327	CF262658
328	TC9918 homologue to emb|X59733.1|XB28SRRNA X.borealis 28S ribosomal RNA gene for 28S rRNA
329	CV994596
330	CF263287
331	CK417182
332	CF263425
333	BM494881
334	BM495311
335	CV994144
336	CF263265
337	CK415622
338	CV991253
339	CV989391
340	CK414829
341	CK410283
342	CF262823
343	TC13839 similar to GB|AAH61475.1|38181905|BC061475 D6Ertd538e protein {Mus musculus;}
344	CK413565
345	CK418345
346	TC12064
347	CV992387
348	CK426178
349	CB936601
350	TC10613 similar to GB|AAH40802.1|27371005|BC040802 Eprs protein {Mus musculus;} , partial (19%)
351	CV990560
352	CK417775
353	CK417352

354	BM497006
355	BE469200
356	TC12653 UP|Q89418 (Q89418) A83R protein, partial (11%)
357	CK421989
358	CK422033
359	CK411053
360	BM029380
361	CF262234
362	CK414559
363	TC13021 homologue to UP|Q6P043 (Q6P043) Zgc:77696, partial (90%)
364	CK425408
365	CV990586
366	CK414551
367	CK425276
368	TC12513 homologue to UP|Q6IQW1 (Q6IQW1) BMP and activin membrane-bound inhibitor, partial (28%)
369	CB937987
370	CV996410
371	CK416617
372	TC10937 weakly similar to UP|Q9FIL2 (Q9FIL2) Gb|AAD32776.1, partial (12%)
373	CK424574
374	CF262607
375	CV994390
376	CK413597
377	TC11620 similar to UP|Q6DRD1 (Q6DRD1) ATP synthase oligomycin sensitivity conferral protein, complete
378	CK423708
379	TC13047 similar to UP|Q804W1 (Q804W1) Parvalbumin isoform 4b (Zgc:103753 protein), complete
380	CK423577
381	BM028020
382	CK420281
383	CK421087
384	CK415475
385	CK412981
386	BE470123
387	CK417192
388	TC9853 similar to GB|BAA85772.1|6116905|AB033770 thymosin beta {Oncorhynchus mykiss;} , complete
389	TC9981 similar to UP|Q9DEQ2 (Q9DEQ2) Beta thymosin, complete
390	CK415113
391	CK412325
392	TC11682 similar to UP|143B_MOUSE (Q9CQV8) 14-3-3 protein beta/alpha (Protein kinase C inhibitor protein-1)
393	CV996939
394	BE470214
395	TC11534 similar to UP|Q7ZY54 (Q7ZY54) M(2)21ab-prov protein, partial (95%)
396	TC13816 weakly similar to UP|Q6J8F7 (Q6J8F7) Capsid protein, partial (3%)
397	BE212628
398	CK421182
399	TC11027
400	CK409777
401	TC13479
402	TC10270 homologue to UP|Q6TNQ9 (Q6TNQ9) Succinate dehydrogenase complex, subunit A, flavoprotein
403	TC13411 weakly similar to UP|Q6ZQ50 (Q6ZQ50) MKIAA0776 protein (Fragment), partial (26%)
404	CK414295
405	CK419893

406	TC14125 similar to UP|Q6GMK9 (Q6GMK9) Zgc:91855, partial (66%)
407	CV993617
408	CV991166
409	CK414726
410	CF262079
411	TC11571 similar to UP|Q90362 (Q90362) G-crystallin, complete
412	CK426036
413	CK416392
414	CK418701
415	CK415409
416	CV991514
417	BM438442
418	TC11721 weakly similar to UP|HEXB_FELCA (P49614) Beta-hexosaminidase beta chain precursor
419	BE469500
420	CK421578
421	TC12668 similar to UP|Q865L8 (Q865L8) Decay-accelerating factor CD55, partial (10%)
422	CF262529
423	TC10236
424	CK416538
425	CK421756
426	TC13844
427	CK424593
428	CB936582
429	CK422623
430	BM425454
431	BM495343
432	TC10959 weakly similar to UP|Q72EF7 (Q72EF7) Sigma-54 dependent DNA-binding response regulator
433	TC13727 weakly similar to UP|CCDA_CAEEL (P35800) Cuticle collagen dpy-10 precursor
434	BM029579
435	TC10435 similar to UP|GAS5_YEAST (Q08193) Glycolipid anchored surface protein 5 precursor, partial
436	CV995572
437	CK425875
438	TC13711 similar to UP|K6PP_HUMAN (Q01813) 6-phosphofructokinase, type C (Phosphofructokinase 1)
439	CK416875
440	TC11206
441	CK413646
442	CK410524
443	BE470482
444	BM494261
445	CK416733
446	CK415620
447	CB938949
448	CV990906
449	TC14056 UP|Q9IB48 (Q9IB48) Nonclathrin coat protein zeta1-COP, complete
450	CK422904
451	CK415800
452	BE212837
453	CK423089
454	BE469527
455	CF262863
456	TC13026
457	CK424137

458	TC14215
459	CK414346
460	CV988305
461	TC10377 homologue to UP|Q801E8 (Q801E8) Cytosolic malate dehydrogenase A, partial (62%)
462	TC11357 similar to UP|Q90YU0 (Q90YU0) Ribosomal protein L27a, partial (35%)
463	CK422530
464	CK412286
465	CK410497
466	CK421942
467	TC12992 homologue to UP|Q5TSW7 (Q5TSW7) ENSANGP00000028399 (Fragment), partial (4%)
468	BM029425
469	CK417653
470	CV989500
471	BM425473
472	CK419045
473	TC10145 UP|Q6IQ86 (Q6IQ86) Zgc:86903, complete
474	CK423443
475	CK412533
476	TC10166 weakly similar to UP|Q75WB8 (Q75WB8) Chitinase2, partial (23%)
477	CK410597
478	TC12374 similar to UP|Q6E5T6 (Q6E5T6) Claudin 31, partial (20%)
479	CV988828
480	TC11630 homologue to UP|PAB1_BOVIN (P61286) Polyadenylate-binding protein 1
481	BE469443
482	CK410085
483	TC10310 similar to UP|Q92101 (Q92101) Glutathione S-transferase , partial (93%)
484	BE468973
485	TC11100 homologue to UP|Q9DGR7 (Q9DGR7) MKK4, partial (15%)
486	CK424919
487	BE212939
488	TC12322 similar to UP|Q6NYL9 (Q6NYL9) Protein kinase C and casein kinase substrate in neurons 2
489	TC11852 homologue to UP|Q6NZZ0 (Q6NZZ0) Timm17a protein, complete
490	CF263575
491	CK418995
492	TC11554 similar to UP|Q71SZ8 (Q71SZ8) Cytochrome c oxidase subunit VIc, partial (96%)
493	TC13906
494	CK413642
495	CK425841
496	CB937770
497	BM494167
498	TC11801 weakly similar to GB|CAA28379.1|673431|MMADIG precursor adipsin {Mus musculus;}
499	BE213107
500	TC9995 similar to UP|Q6PEI9 (Q6PEI9) Collagen, type I, alpha 3, partial (41%)
501	CK422304
502	CK418186
503	TC11449 similar to UP|Q802Z9 (Q802Z9) Zgc:55489, partial (96%)
504	CK426353
505	CK419272
506	CK414317
507	CK411058
508	CK415291
509	TC11154 similar to UP|Q6IQU2 (Q6IQU2) Zgc:86610, partial (94%)

510	CK416908
511	CK412081
512	CK423709
513	TC12266 similar to UP|Q8YCG1 (Q8YCG1) IOLB PROTEIN, partial (6%)
514	CK413699
515	CK416284
516	BE468724
517	TC10642 similar to UP|Q6P0U7 (Q6P0U7) Zgc:77730 protein, partial (25%)
518	TC13593 similar to UP|Q5XJP4 (Q5XJP4) Zgc:101710, partial (95%)
519	CK421749
520	CK422565
521	BM494355
522	TC11962 similar to UP|Q6GQM3 (Q6GQM3) Proteasome (Prosome, macropain) subunit, alpha type, 6b
523	CK415720
524	CV991806
525	CK415981
526	CF263123
527	CK421272
528	BE212725
529	CK420733
530	BM027998
531	CK416576
532	TC13947 homologue to UP|Q26165 (Q26165) Beta tubulin, partial (49%)
533	TC12586 similar to UP|Q8I727 (Q8I727) TcC31.32, partial (9%)
534	TC14137 weakly similar to UP|Q9I933 (Q9I933) Complement C4-1, partial (5%)
535	TC11700 homologue to UP|Q6DI14 (Q6DI14) Zgc:86841, partial (97%)
536	TC11728 similar to UP|Q8WY42 (Q8WY42) Splicing-related factor RNPS1, complete
537	CK420426
538	BM438197
539	CK412787
540	CK418013
541	CB940530
542	CK422383
543	TC10926
544	CK420633
545	TC13447
546	CK422657
547	BM496460
548	CV995745
549	TC10733 similar to GB|CAA46992.1|63803|GGTENS tensin {Gallus gallus;} , partial (74%)
550	CV989527
551	TC12918 homologue to UP|Q90YU0 (Q90YU0) Ribosomal protein L27a, complete
552	TC10050 similar to UP|BHB2_HUMAN (O14503) Class B basic helix-loop-helix protein 2 (bHLHB2)
553	TC12455
554	TC13018 homologue to UP|Q7T2A8 (Q7T2A8) SH3 domain binding glutamic acid-rich protein like, complete
555	CK417940
556	CK414352
557	CK417953
558	CK415447
559	TC9872 similar to UP|O62896 (O62896) MHC class I alpha chain, partial (92%)
560	TC13508
561	TC11649 homologue to PIR|A31486|A31486 translation initiation factor eIF-5A [validated] - rabbit

562	CB940020
563	BM028956
564	CK417445
565	TC10213 homologue to UP|Q9N178 (Q9N178) Type X collagen, partial (3%)
566	CB940562
567	TC14028 similar to UP|Q6IQG6 (Q6IQG6) Hprt1 protein, partial (93%)
568	CK417457
569	TC10304 similar to UP|Q922L2 (Q922L2) Apoptosis inhibitor 5, partial (63%)
570	CK416950
571	CK413545
572	CF262312
573	CF262283
574	CK419326
575	TC11427
576	TC12246 homologue to UP|Q6PGZ8 (Q6PGZ8) Xab2 protein, partial (41%)
577	CK420762
578	CV989458
579	CF262254
580	CK415487
581	TC12016 similar to UP|PEN2_BRARE (Q8JHF0) Gamma-secretase subunit Pen-2
582	CK410382
583	CK418895
584	CK412917
585	CK421403
586	TC11844 UP|Q6TXI9 (Q6TXI9) LRRGT00010, partial (13%)
587	CK424066
588	CF261762
589	CK421327
590	CK415932
591	CV991700
592	TC13168 UP|Q64150 (Q64150) Nuclear localization signal binding protein, partial (15%)
593	TC11858 similar to UP|LPP1_CAVPO (O88956) Lipid phosphate phosphohydrolase 1
594	TC10241 homologue to UP|Q6PH06 (Q6PH06) Zgc:63469, partial (28%)
595	TC11920
596	CK416378
597	TC12368 weakly similar to UP|Q6P2I4 (Q6P2I4) CSGlcA-T protein, partial (35%)
598	CF262214
599	CK418588
600	CK423077
601	TC10977 homologue to UP|Q70V42 (Q70V42) Small inducible cytokine B14, complete
602	CK420354
603	BM439086
604	CK410000
605	TC12323
606	TC13685 similar to GB|AAC50902.1|1732075|HSU75309 TBP-associated factor {Homo sapiens;} , partial
607	CK417867
608	CK423027
609	CV996288
610	CK417206
611	CK419741
612	CV989790
613	CV994752

614	CK421855
615	CF262107
616	CK416917
617	BM495392
618	CK423611
619	BM494127
620	BE470257
621	CK413375
622	CK413346
623	BM495935
624	TC11405 homologue to UP|Q6IQX1 (Q6IQX1) Myosin, heavy polypeptide 2, fast muscle specific, partial
625	CK416721
626	TC12061
627	CV987397
628	TC11218 similar to UP|GNPI_HUMAN (P46926) Glucosamine-6-phosphate isomerase
629	CF262100
630	BE468360
631	CK415427
632	CK425916
633	CF263523
634	TC11629 similar to UP|Q5U3R7 (Q5U3R7) Zgc:101706, partial (57%)
635	TC14114 similar to UP|ATPD_BOVIN (P05630) ATP synthase delta chain, mitochondrial precursor
636	CK413474
637	BM438645
638	CK423208
639	CK415965
640	TC13370 UP|Q8UWK5 (Q8UWK5) Novel immune-type receptor 10, complete
641	CK423965
642	CV991331
643	CV993394
644	CK415728
645	BM438893
646	CK420892
647	BM494399
648	TC12632
649	CK411705
650	BE468535
651	CK415296
652	TC13133
653	CK422123
654	CK416846
655	TC9851 similar to UP|EPCR_BOVIN (Q28105) Endothelial protein C receptor precursor
656	TC11055 similar to UP|Q63ZM4 (Q63ZM4) Armet protein, partial (92%)
657	TC10568 homologue to GB|AAC09367.1|3005758|AF055028 RNA polymerase II 140 kDa subunit {Homo sapiens}
658	CK413782
659	CF262619
660	CK418743
661	CK415521
662	CK415914
663	CK425717
664	CF263155
665	TC11203 similar to GB|AAH02920.1|12804133|BC002920 DC-TM4F2 protein {Homo sapiens;} , partial (60%)

666	CK411876
667	BE469478
668	TC10005 weakly similar to UP|Q98SS1 (Q98SS1) Egg envelope protein ZP2 variant C, partial (73%)
669	BM029369
670	CK411349
671	CB940024
672	CF262870
673	CK418857
674	TC13116 similar to UP|CLDY_BRARE (Q9YH91) Claudin-like protein ZF-A89, partial (97%)
675	TC12857 similar to UP|ATA1_MAKNI (P70083) Sarcoplasmic/endoplasmic reticulum calcium ATPase 1
676	CK417433
677	TC10124 weakly similar to UP|IBP7_HUMAN (Q16270) Insulin-like growth factor binding protein 7 precursor
678	TC14194 UP|RL41_CYPCA (Q6YLX4) 60S ribosomal protein L41, partial (72%)
679	CK417885
680	BE470181
681	CV991178
682	TC11446
683	CV992622
684	CK409752
685	TC13561
686	BE468927
687	TC11856 homologue to UP|Q9Z1W5 (Q9Z1W5) Stress-associated endoplasmic reticulum protein 1 (Mus musculus)
688	CB940799
689	CV993994
690	CK411609
691	CK415182
692	CK412572
693	TC11761 homologue to UP|Q7SXS5 (Q7SXS5) Zgc:63844, complete
694	TC10271 similar to UP|Q8CHA2 (Q8CHA2) MKIAA1463 protein (Fragment), partial (54%)
695	BE470280
696	CK414672
697	CB937869
698	CK413032
699	CK416673
700	CV989869
701	CK418231
702	TC11694 homologue to UP|Q7T2P2 (Q7T2P2) Chaperonin containing TCP1, subunit 3 (Gamma)
703	TC11327 UP|Q7ZVK2 (Q7ZVK2) Ubiquitin-conjugating enzyme E2G 1, complete
704	TC13811 similar to UP|Q7TMM3 (Q7TMM3) Arl6ip2 protein, partial (67%)
705	CV995877
706	CV993679
707	TC12109 homologue to UP|Q9BU59 (Q9BU59) Smu-1 suppressor of mec-8 and unc-52 homolog
708	TC10013 UP|Q90YV9 (Q90YV9) Ribosomal protein L10, complete
709	CK414898
710	BM438578
711	TC13872 similar to UP|Q6DHN6 (Q6DHN6) Zgc:92198, partial (48%)
712	TC11984 similar to UP|Q8UUT4 (Q8UUT4) DMbeta1 (Glycoprotein M6Aa), partial (77%)
713	BM494674
714	CK411239
715	CV988969
716	CK424328
717	CV988772

718	TC11683 UP|Q8R4X0 (Q8R4X0) Fibroblast growth factor-like factor-4D (Fragment), partial (6%)
719	CK416229
720	CK423587
721	TC11961 homologue to UP|Q7S1G6 (Q7S1G6) Predicted protein, partial (5%)
722	CK418305
723	BE212985
724	CV995070
725	TC11894 homologue to UP|Q7ZVZ4 (Q7ZVZ4) Regulator of nonsense transcripts 1, partial (25%)
726	CK410790
727	TC14020
728	CK409724
729	TC9903 similar to UP|Q6DF51 (Q6DF51) HUS1 checkpoint homolog, partial (84%)
730	CK410197
731	BE469698
732	BM028060
733	CV990614
734	CK412023
735	TC13039
736	CV988811
737	CV995738
738	CV993196
739	TC11608 weakly similar to UP|APA1_BRARE (O42363) Apolipoprotein A-I precursor (Apo-AI) (ApoA-I)
740	CK417036
741	TC12996 homologue to UP|O57150 (O57150) H88, partial (30%)
742	TC10958 similar to UP|ITB1_CHICK (P07228) Integrin beta-1 precursor (CSAT antigen) (JG22 antigen)
743	CV989517
744	TC12004
745	CK420077
746	CK410714
747	CK413660
748	CK418479
749	TC9912 similar to UP|Q6TNV0 (Q6TNV0) Cytochrome c oxidase subunit IV isoform 1, complete
750	CK414943
751	CK423335
752	TC11559 homologue to UP|Q7SXT9 (Q7SXT9) Eukaryotic translation initiation factor 2 gamma, complete
753	CK417367
754	CK421511
755	CB939526
756	CV990183
757	CF263074
758	CK419874
759	CB937095
760	CV994510
761	TC13787
762	TC13173 homologue to UP|Q7T385 (Q7T385) ATPase, H+ transporting, lysosomal, V1 subunit C, isoform 1
763	CK420312
764	CF263195
765	CK416401
766	TC12098 homologue to UP|Q803C2 (Q803C2) Db83 protein, partial (98%)
767	TC10066 GB|AAK95190.1|15294027|AF402816 40S ribosomal protein S8 {Ictalurus punctatus;} , complete
768	CK422172
769	BM425070

770	BM496409
771	TC11862 homologue to UP|Q6IQH4 (Q6IQH4) Zgc:86762, partial (97%)
772	CK413689
773	TC10564 similar to UP|Q9V3Y5 (Q9V3Y5) CG4119-PA (BcDNA.LD23634), partial (5%)
774	CB939288
775	TC13588 similar to UP|ROM_MOUSE (Q9D0E1) Heterogeneous nuclear ribonucleoprotein M (hnRNP M)
776	BM424909
777	CK414407
778	TC10877 homologue to UP|Q6IMK3 (Q6IMK3) Rbj, partial (46%)
779	BM028933
780	CV993045
781	TC14124 similar to GB|AAC16002.1|3126878|AF061832 M4 protein deletion mutant {Homo sapiens;}
782	CK422106
783	CK412704
784	TC10034 similar to UP|O73911 (O73911) K123 protein precursor, partial (10%)
785	CK411961
786	TC12757
787	CK415717
788	CK411287
789	TC11643 similar to UP|Q6IQ59 (Q6IQ59) Ubiquinol-cytochrome c reductase core protein II, partial
790	CV991583
791	CK420612
792	CV987722
793	BM425409
794	CK419566
795	CK419265
796	CK414505
797	TC12112 similar to UP|Q6DRK5 (Q6DRK5) Cotamer alpha, partial (44%)
798	CB939259
799	CK418299
800	CK419145
801	CK420715
802	CK410042
803	CK421258
804	CK425198
805	CK420787
806	TC13009 similar to UP|Q804G7 (Q804G7) Annexin 4 (Annexin A4), complete
807	CV989758
808	TC11669 homologue to UP|Q6TLG9 (Q6TLG9) Protein tyrosine phosphatase type IVA, member 2, partial
809	CK414409
810	CK418281
811	CK417029
812	CK425439
813	BM425097
814	BE470468
815	CK412463
816	CK414533
817	CK415405
818	TC10549 similar to UP|Q629V3 (Q629V3) 2-oxoisovalerate dehydrogenase, E2 component, dihydrolipamide
819	TC10892 similar to UP|Q15598 (Q15598) Titin (Fragment), partial (5%)
820	CK418688
821	TC13434

822 CK419980
823 BM494700
824 CV994153
825 CK418380
826 CK414093
827 CK425278
828 CK412199
829 CF262063
830 CK419066
831 CF263237
832 CK415594
833 TC14090 similar to UP|Q719N3 (Q719N3) Macrophage/microglia activation-associated factor (Maf protein)
834 TC14148
835 TC12939 UP|O62895 (O62895) MHC class I alpha chain, complete
836 CK423194
837 TC10913 UP|Q64150 (Q64150) Nuclear localization signal binding protein, partial (8%)
838 TC12244 weakly similar to UP|RT29_HUMAN (P51398) Mitochondrial 28S ribosomal protein S29 (S29mt)
839 BM495124
840 TC12650 weakly similar to GB|AAL28433.1|16768428|AY060885 GM04427p {Drosophila melanogaster;}
841 TC10139 UP|P2AB_RABIT (P11611) Serine/threonine protein phosphatase 2A, catalytic subunit, beta isoform
842 BM495074
843 CK413355
844 CK421561
845 TC12548
846 CK424281
847 CV993604
848 CK421324
849 BM029499
850 TC10389 weakly similar to UP|Q9W6G2 (Q9W6G2) Fertilization envelope outer layer protein, partial
851 TC12563 weakly similar to GB|AAC51775.2|21903712|HSU65090 carboxypeptidase D {Homo sapiens;}
852 CV987774
853 CK412666
854 BM424721
855 CV992474
856 CK414492
857 BM439102
858 BE470403
859 TC10098 similar to UP|Q7T2N0 (Q7T2N0) Interferon-inducible protein Gig2, partial (91%)
860 CF263513
861 TC13730 homologue to UP|Q6DRI2 (Q6DRI2) Clatherin heavy chain, partial (31%)
862 CF263639
863 CK413069
864 TC13196 homologue to UP|Q9I9E9 (Q9I9E9) EIF2 alpha subunit (Eukaryotic translation initiation factor 2)
865 TC10758 similar to UP|Q8MS43 (Q8MS43) RE09079p, partial (3%)
866 CK415474
867 BE468292
868 TC12660 similar to UP|Q7SZQ8 (Q7SZQ8) Telomerase binding protein, p23, partial (83%)
869 TC13962 similar to UP|Q8QGB2 (Q8QGB2) VHSV-induced protein-6, partial (55%)
870 CF263538
871 TC11655 homologue to UP|O42447 (O42447) Id1 protein, partial (91%)
872 TC12764 similar to UP|Q63647 (Q63647) Proline rich protein, partial (15%)
873 CK426167

874	CK422162
875	CK421105
876	TC13061 homologue to UP|Q6DGZ5 (Q6DGZ5) Zgc:92709, partial (51%)
877	CK426149
878	TC13125 weakly similar to UP|Q9WUJ3 (Q9WUJ3) Myomegalin, partial (3%)
879	CK409841
880	CV990296
881	CK420547
882	TC11869 UP|Q672Z0 (Q672Z0) CC chemokine SCYA102, complete
883	CK417324
884	CK413250
885	CK415188
886	CV994607
887	TC10956 similar to UP|Q6DGT6 (Q6DGT6) Zgc:92778, partial (75%)
888	TC10731 similar to UP|Q6NZ32 (Q6NZ32) Zgc:77439 (Dimethylanaline monooxygenase-like), partial (53%)
889	CK424320
890	CK425385
891	TC13278 similar to UP|Q8UVQ5 (Q8UVQ5) X-box binding protein 1A (X-box binding protein 1), partial
892	CK413045
893	CK417241
894	CK414428
895	TC10995
896	TC10621 homologue to UP|Q7ZVP2 (Q7ZVP2) RAB5C, member RAS oncogene family, partial (92%)
897	BM494934
898	TC13732
899	CK414323
900	CK416201
901	CV995380
902	TC13686 OMNI|NTL01PA0883|CAB49807.1|5458318| desulfoferrodoxin {Pyrococcus abyssi;} , partial (8%)
903	TC11080 similar to PIR|A54021|A54021 phosphotyrosyl phosphatase activator PTPA - human {Homo sapiens}
904	TC12242 similar to UP|RCAS_MOUSE (Q9D0V7) Receptor-binding cancer antigen expressed on SiSo cells
905	TC10936
906	TC12956 UP|Q6YNX6 (Q6YNX6) Calmodulin 2, complete
907	TC10572 similar to UP|Q7T028 (Q7T028) GDP-fucose protein O-fucosyltransferase 1 , partial (29%)
908	TC10659
909	CK424015
910	CF262038
911	CK416138
912	CV988531
913	CV990686
914	TC13332 weakly similar to GB|AAC51784.1|2507613|HSU75329 serine protease {Homo sapiens;} , partial
915	TC10288 homologue to UP|O57513 (O57513) Serine/threonine protein kinase, partial (32%)
916	CK416793
917	CK413757
918	CK414239
919	CV990666
920	CK417461
921	TC13131 similar to UP|Q80XP4 (Q80XP4) Uap1l1 protein (Fragment), partial (74%)
922	CK416370
923	CK423502
924	TC12335 UP|Q8DNE2 (Q8DNE2) Acylphosphate phosphohydrolase , partial (11%)
925	TC10687 similar to UP|Q66KC4 (Q66KC4) MGC89719 protein, partial (44%)

926	CK420760
927	TC10699 homologue to UP|Q6NSM5 (Q6NSM5) Zgc:85981, partial (52%)
928	CK416227
929	CK424427
930	TC13826 homologue to UP|P300_HUMAN (Q09472) E1A-associated protein p300 , partial (3%)
931	TC9962 similar to UP|Q6P3K5 (Q6P3K5) Ckii protein, partial (42%)
932	TC11952 similar to UP|Q5W0B9 (Q5W0B9) TBC1 domain family, member 4, partial (8%)
933	TC11419 weakly similar to UP|Q90WF7 (Q90WF7) Warm-temperature-acclimation-related-65 kDa-protein protein
934	CK413752
935	TC12974 similar to UP|MOT3_YEAST (P54785) Zinc finger protein MOT3/HMS1, partial (6%)
936	CK423456
937	CK414783
938	TC9937 homologue to UP|Q6PBW6 (Q6PBW6) Chaperonin containing TCP1, subunit 2 (Beta), complete
939	CK423200
940	CK426220
941	TC9891 homologue to UP|RBM8_HUMAN (Q9Y5S9) RNA-binding protein 8A (RNA binding motif protein 8A)
942	CK412815
943	CV988285
944	CK426305
945	TC11297 similar to GB|BAA92493.1|7229115|AB036524S9 ribonucleotide reductase {Homo sapiens;}
946	TC12936 UP|Q6ZWL8 (Q6ZWL8) Mus musculus 7 days embryo nullipotent stem cell CRL-2070 NE cDNA, RIKEN
947	TC10719 homologue to UP|Q7T3E6 (Q7T3E6) Bmi1b protein, partial (56%)
948	CK424615
949	TC10393 similar to UP|Q6RIA7 (Q6RIA7) 14-alpha demethylase, partial (38%)
950	CV991835
951	CK423322
952	BM027990
953	BE212865
954	TC13090 UP|Q6E5U7 (Q6E5U7) Fas receptor, complete
955	CV988152
956	TC12558 weakly similar to UP|Q7T2N0 (Q7T2N0) Interferon-inducible protein Gig2, partial (73%)
957	BE213054
958	TC13051 similar to UP|Q8AV10 (Q8AV10) Sdf1a (Chemokine ligand 12), partial (78%)
959	TC10645 homologue to UP|Q6NY88 (Q6NY88) Zgc:76972, partial (92%)
960	CK414174
961	CB938337
962	CK422701
